# Metabolism of the predominant human milk oligosaccharide fucosyllactose by an infant gut commensal

**DOI:** 10.1038/s41598-019-51901-7

**Published:** 2019-10-28

**Authors:** Kieran James, Francesca Bottacini, Jose Ivan Serrano Contreras, Mariane Vigoureux, Muireann Egan, Mary O’connell Motherway, Elaine Holmes, Douwe van Sinderen

**Affiliations:** 1 APC Microbiome Ireland, University College Cork, Western Road, Cork, Ireland; 2 School of Microbiology, University College Cork, Western Road, Cork, Ireland; 3 Department of Surgery and Cancer, Imperial College London, South Kensington, London SW7 2AZ United Kingdom; 4 The Centre for Computational and Systems Medicine, Health Futures Institute, Murdoch University, Harry Perkins Institute of Medical Research, 5 Robin Warren Drive, Perth, WA 6150 Australia

**Keywords:** Cellular microbiology, Bacterial genomics

## Abstract

A number of bifidobacterial species are found at a particularly high prevalence and abundance in faecal samples of healthy breastfed infants, a phenomenon that is believed to be, at least partially, due to the ability of bifidobacteria to metabolize Human Milk Oligosaccharides (HMOs). In the current study, we isolated a novel strain of *Bifidobacterium kashiwanohense*, named APCKJ1, from the faeces of a four-week old breastfed infant, based on the ability of the strain to utilise the HMO component fucosyllactose. We then determined the full genome sequence of this strain, and employed the generated data to analyze fucosyllactose metabolism in *B. kashiwanohense* APCKJ1. Transcriptomic and growth analyses, combined with metabolite analysis, *in vitro* hydrolysis assays and heterologous expression, allowed us to elucidate the pathway for fucosyllactose metabolism in *B. kashiwanohense* APCKJ1. Homologs of the key genes for this metabolic pathway were identified in particular in infant-derived members of the *Bifdobacterium* genus, revealing the apparent niche-specific nature of this pathway, and allowing a broad perspective on bifidobacterial fucosyllactose and L-fucose metabolism.

## Introduction

Breast-feeding influences neonatal intestinal microbiota development and composition^1–3^, with significant differences reported between the gut microbiota of breast-fed infants and their formula-fed counterparts^4^. It currently is believed that Human Milk Oligosaccharides (HMOs) represent some of the most influential components of human breastmilk and colostrum in terms of their impact on microbiota composition by acting as a selective growth substrate^2,3,5^, with recent work correlating differences in the neonatal microbiota with variations in HMO composition of corresponding mothers milk^6^. After lactose, HMOs are the most abundant carbohydrate component of breastmilk^2,7^, and are composed of a heterogeneous mix of at least 200 distinct glycan structures^8^. HMOs consist of a lactose moiety linked, through a variety of different glycosidic connections, to L-fucose, sialic acid, lacto-N-biose (LNB) or N-acetyllactosamine (LacNAc); the latter two cases creating the tetrasaccharides lacto-N-tetraose (LNT) or lacto-N-neotetraose (LNnT), respectively. LN(n)T may be elongated by additional LNB or LacNAc residues at the reducing end, and/or may be fucosylated or sialylated at different positions^2,8,9^. HMOs can be viewed as a model prebiotic, since it complies with the definition of the latter as a ‘non-digestible food ingredient that beneficially affects the host by selectively stimulating growth and/or activity of one or a limited number of bacteria in the colon, thereby improving host health^10^. Recent studies have furthermore shown the beneficial properties of HMOs extending to protection against infant-associated gastrointestinal conditions, such as *Campylobacter* and Group B Streptococcal infections, and necrotising enterocolitis (NEC)^11^.

Bifidobacteria are Gram-positive, anaerobes belonging to the Actinobacteria phylum, and are commonly observed as commensal members of the mammalian, avian and insect intestinal tract^12^. Enrichment of bifidobacteria has been observed in the faecal microbiota of breastfed infants^13^, to whom they are believed to provide an array of health benefits^5,14–16^. The dominance of bifidobacteria in this niche is attributed to the presence of a small number of key species, which are capable of selectively utilising (particular) HMOs as their sole carbohydrate source^5,8,17,18^. These infant-associated species include *Bifidobacterium bifidum*, *Bifidobacterium longum* subsp. *infantis* and *Bifidobacterium breve*. *B. bifidum* carries out extracellular hydrolysis of complex HMOs, including LN(n)T, and fucosylated and sialylated structures, prior to the import and metabolism of (many of) the resultant mono- and di-saccharides^19–25^. *B. breve* and *B. longum* subsp. *infantis* internalise (certain) intact HMO moieties, such as LNT, LNnT and LNB, and sequentially degrade them for subsequent metabolism of the generated monosaccharides^8,19,26–30^. Furthermore, *B. infantis* internalizes and then metabolizes various fucosylated and sialylated HMOs^31–33^.

*Bifidobacterium kashiwanohense* has been previously isolated from the faeces of neonates^34,35^, though it is not considered a common component of the neonatal gut microbiota. There is little knowledge of this species and its metabolic capabilities. The ability of two *B. kashiwanohense* strains to grow on the HMO components 2′-fucosyllactose (2′-FL) and 3-fucosyllactose (3-FL) was recently shown, although these strains did not appear to metabolize the (fucosyllactose component) L-fucose^33^. While the presence of fucosylated HMOs in breastmilk has been shown to vary greatly, depending on genetic and geographical factors^36^, fucosylated HMOs are generally thought to represent ~50–80% of all HMOs found in human breastmilk^37^. Furthermore, free 2′-FL and 3-FL represent between 12–45% and 0.5–3% of the total HMO content, respectively^36^. 2′-FL (Fucα1-2[Galβ1-4]Glc) consists of L-fucose linked by an α1-2 bond to the galactose residue in lactose, while its isomer 3-FL (Fucα1-3Galβ1-4Glc) consists of L-fucose linked by an α1-3 bond to the glucose moiety in lactose^37^. The ability to metabolize 2′-FL and/or 3-FL is therefore considered to be important for infant gut colonisation^38^. Of note, FL-utilising properties of certain members of bifidobacterial species have a crucial impact on metabolite production and early life gut microbiota composition^38^. Metabolite profiles for *B. kashiwanohense* following growth on 2′-FL or 3-FL suggest that the inability of *B. kashiwanohense* to grow on L-fucose is due to the lack of two enzymes, L-fucose mutarotase and fucose permease^33^. The latter study, and another recent study^39^, however, demonstrated L-fucose metabolism resulting in the formation of 1,2-propanediol (1,2-PD), during growth on fucosyllactose by *B. breve, B. longum* subsp. *infantis* and *B. longum* subsp. *suis*, which suggests the presence of a common pathway for L-fucose metabolism. Notably, trophic interactions between the butyrate-producing gut commensal *Eubacterium hallii* and *B. breve* and/or *B. longum* subsp. *infantis* driven by FL and L-fucose utilisation have recently been shown to occur in the gut of infants^39^. Importantly, *E. hallii* represents one of the first producers of butyrate in the infant gut, while it is also able to convert 1,2-PD to propionate^40^.

In the current study, we sequenced the genome of *B. kashiwanohense* strain APCKJ1, isolated from faeces of a breastfed infant using 2′-FL as a selective carbohydrate. Employing various approaches we dissected fucosyllactose metabolism in APCKJ1, and also identified key genes involved in L-fucose metabolism in *B. breve*. Subsequent bioinformatic analysis revealed the distribution of fucose/fucosyllactose utilisation genes among members of the genus *Bifidobacterium*. This work not only elucidates details of fucosyllactose metabolism in *B. kashiwanohense*, but also allows general insights into bifidobacterial L-fucose metabolism.

## Results

### Isolation of a novel *B. kashiwanohense* isolate

A single stool sample from an exclusively breast-fed neonate, aged 4 weeks, was employed to isolate 2′-FL-utilising *Bifidobacterium* isolates. Several *Bifidobacterium* isolates were obtained from this faecal sample, based on their resistance to mupirocin (which selects for bifidobacteria^41,42^) and ability to grow on 2′-FL as the sole carbohydrate source. All assessed isolates were found to belong to the species *B. kashiwanohense* and appeared to be clonal. As this is an infrequently encountered species (from infant faecal samples), yet clearly exhibiting an ability to grow on 2′-FL, one of these isolates, designated APCKJ1, was selected for further characterisation.

### Carbohydrate utilisation by *B. kashiwanohense* APCKJ1

**Figure 1:**
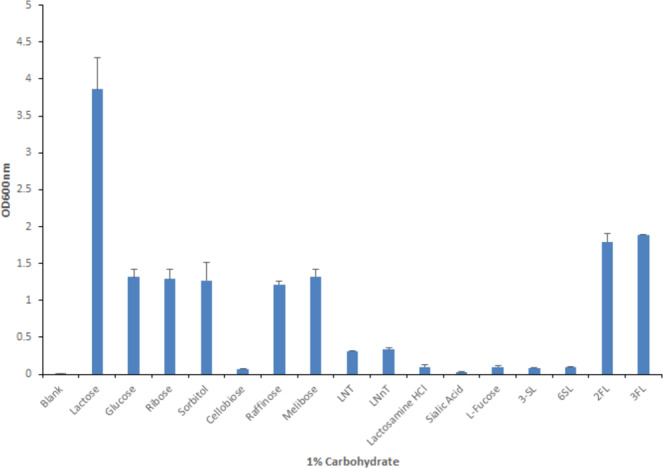
Carbohydrate utilization profiles of *B. kashiwanohense* APCKJ1. Final OD_600nm_ values after 24 hours of growth of *B. kashiwanohense* APCKJ1 in modified MRS containing a range of carbohydrates at 1% (wt/vol) as the sole carbon source. The results are mean values obtained from two separate experiments. Error bars represent the standard deviation. Figure adapted from thesis Figure 5.1; James, 2018^97^.

In order to further investigate the carbohydrate utilisation capabilities of *B. kashiwanohense* APCKJ1, growth assays were carried out for this strain inoculated in modified de Man Rogosa and Sharpe (mMRS) medium supplemented with 1% (wt/vol) of one of 16 different sugars, including nine HMOs or HMO-components (Fig. 1). Growth was assessed by measuring the OD_600nm_ following 24 hours of anaerobic growth at 37 °C. APCKJ1 was shown to reach its highest level of cell density following growth on lactose (OD_600nm_ > 3.0), while also reaching high optical densities following growth on the monosaccharides glucose, ribose, sorbitol, as well as the disaccharide melibiose and the trisaccharide raffinose. Good growth (final OD_600nm_ > 1.5) was observed for just two of the nine HMO components tested: the isomers 2′-FL and 3-FL. This confirmed the observation that APCKJ1 is capable of metabolising 2′-FL as its sole carbohydrate source. L-fucose, however, did not support growth of APCKJ1 to any substantial degree, suggesting the strain is incapable of metabolizing this sugar when present in the growth medium in its free form.

**Figure 2:**
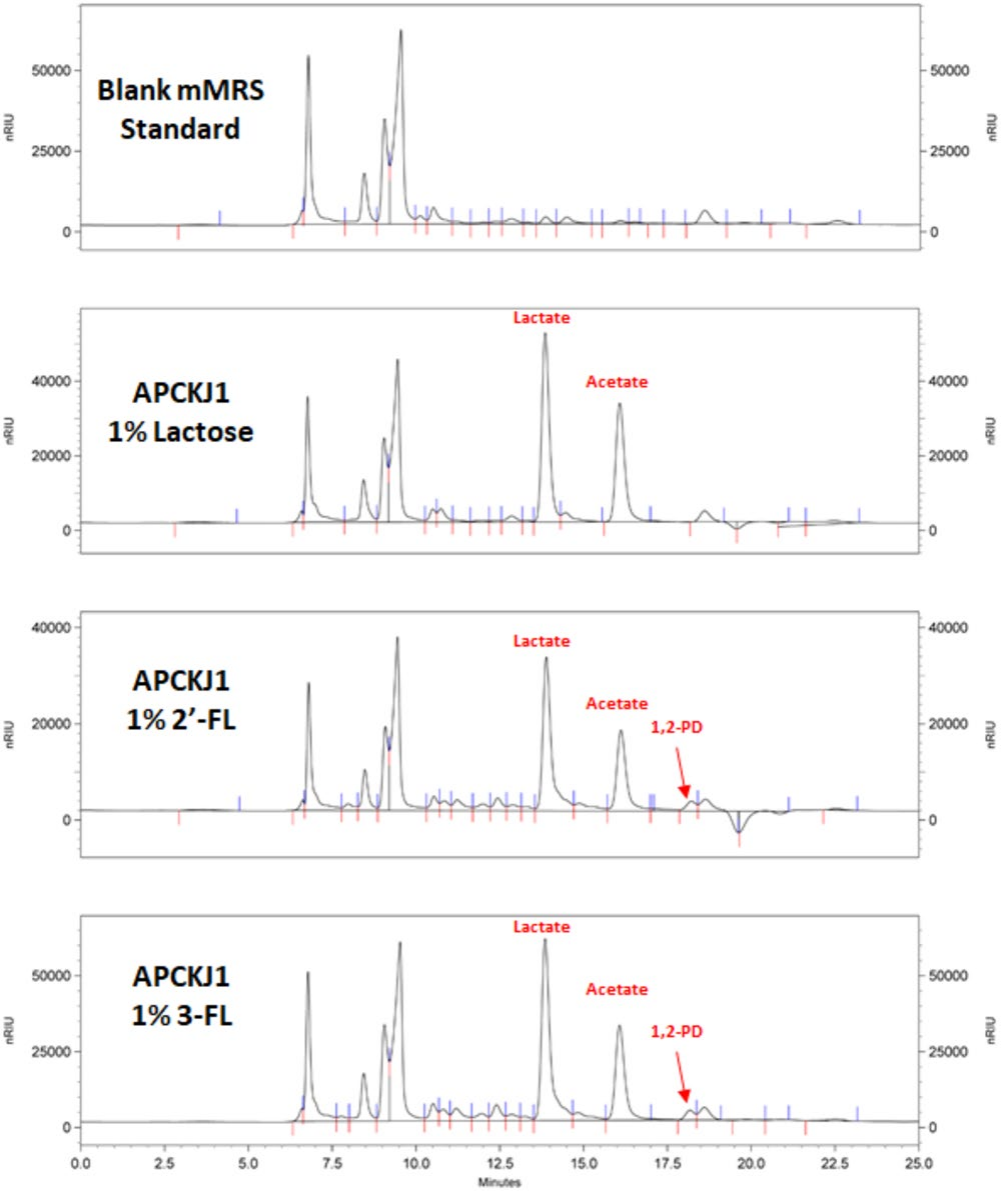
Fermentation of lactose, 2′-FL and 3-FL by *B. kashiwanohense* APCKJ1. HPLC chromatogram profiles of fermentations of mMRS containing 1% lactose, 1% 2′-FL or 1% 3-FL inoculated with *B. kashiwanohense* APCKJ1, following 24 hours growth anaerobically at 37 °C, as well as a blank mMRS control. Figure adapted from thesis Figure 5.2; James, 2018^97^.

The cell-free supernatants of APCKJ1 following growth on 2′-FL or 3-FL (or the control carbohydrate lactose) were analysed by High Performance Liquid Chromatography (HPLC) for the presence of specific metabolic end-products (Fig. 2). As additional controls we also analysed uninoculated mMRS containing lactose, 2′-FL, 3-FL or no carbohydrate source. This analysis revealed that growth of *B. kashiwanohense* APCKJ1 on either 2′-FL or 3-FL causes the accumulation of lactate and acetate in the growth medium (Fig. 2), which are known metabolic end products of lactose and fucosyllactose metabolism in bifidobacteria^33,39,43^. Furthermore, a peak was observed corresponding to 1,2-PD, which has been put forward as an end product of L-fucose metabolism^33,39^. These findings indicate that *B. kashiwanohense* APCKJ1 is not only capable of degrading 2′-FL/3-FL and metabolizing its lactose component, but that it also utilises the L-fucose constituent of these HMOs.

### General genome features of *B. kashiwanohense* APCKJ1, and transcriptome analysis during growth on 2′-FL or 3-FL

To identify the genes involved in 2′-FL/3-FL metabolism we first sequenced the genome of *B. kashiwanohense* isolate APCKJ1, revealing a 2,445,409-base pair (bp) circular molecule, containing 53 tRNA genes and 5 rRNA operons. The G + C content of the genome is 56.19%, with a total of 2,110 predicted protein encoding sequences (CDS). Scores for the sequencing reads and quality are given in Table S1. We then assessed global gene expression by microarray analysis of *B. kashiwanohense* APCKJ1 when grown in mMRS supplemented with 2′-FL, and compared with the transcriptome obtained during growth in mMRS supplemented with sorbitol or with lactose. Genes that were shown to be significantly up- or down-regulated in transcription above the designated cut-off (fold-change > 5.0, P < 0.001) are shown in Table 1. A fold-change of 5.0 was chosen as the threshold for differential expression, as this allowed a clear resolution between genes that exhibit a high level of increased transcription and (a large number of) genes eliciting a lower level of increased transcription, which was presumed to be due to background variation. When grown on 2′-FL (and compared to the transcriptome of the strain when grown on sorbitol or lactose), the transcriptionally upregulated genes of strain APCKJ1 included all genes (with the exception of a predicted regulator, *fumR*) of a gene cluster corresponding to locus tags BKKJ1_2069-2079 (and designated here as the *fum* locus [for fucose metabolism]) (Table 1 and Fig. S1). This gene cluster was found to contain two predicted and adjacent α-fucosidase-encoding genes, BKKJ1_2069 and BKKJ1_2070 (designated *fumA1* and *fumA2*, respectively). Microarray analysis also revealed that transcription of *lacA*, encoding a predicted β-galactosidase, and the adjacent *lacS*, encoding a predicted lactose permease, was increased when cells were grown in a medium containing 2′-FL and compared to transcription patterns when grown on sorbitol or lactose, respectively. The *lacA* and *lacS* gene cluster correspond to locus tags BKKJ1_2066 and 2067, respectively, and are located adjacent to the *fum* cluster. Based on their annotations, the *lac* genes and the *fum* gene cluster are predicted to function in lactose and fucosyllactose/L-fucose metabolism, respectively.

**Table 1:** *B. kashiwanohense* APCKJ1 genes that are transcriptionally upregulated during growth in mMRS medium supplemented with 2′-FL as the sole carbohydrate, as compared to growth in mMRS supplemented with sorbitol.

Gene ID	Gene name	Function	2′-FL vs Sorbitol	2′-FL vs Lactose
BKKJ1_0067	BKKJ1_0067	ABC transporter substrate-binding protein	−8.12	—
BKKJ1_0068	BKKJ1_0068	ABC transporter permease	−5.91	—
BKKJ1_0069	BKKJ1_0069	ABC transporter permease	−5.71	—
BKKJ1_0206	BKKJ1_0206	6-phosphogluconate dehydrogenase	−13.31	—
BKKJ1_0207	BKKJ1_0207	hypothetical protein	−17.67	—
BKKJ1_0208	BKKJ1_0208	putative gluconokinase	−17.21	—
BKKJ1_0336	BKKJ1_0336	hypothetical protein	−16.99	—
BKKJ1_0338	BKKJ1_0338	hypothetical protein	−27.69	—
BKKJ1_0339	BKKJ1_0339	xylitol (sorbitol) dehydrogenase	−34.61	—
BKKJ1_0340	BKKJ1_0340	transcriptional regulator	−36.34	—
BKKJ1_0341	BKKJ1_0341	aldehyde-alcohol dehydrogenase 2	−22.03	—
BKKJ1_0429	*fumG*	L-1,2-propanediol oxidoreductase	—	5.90
BKKJ1_2066	*lacS*	galactoside symporter	—	5.35
BKKJ1_2067	*lacA*	beta-galactosidase	6.47	—
BKKJ1_2068	*lacR*	Truncated LacI-type transcriptional regulator	—	—
BKKJ1_2069	*fumA1*	GH95 alpha-1 3/4-fucosidase	7.45	6.88
BKKJ1_2070	*fumA2*	GH29 alpha-1 3/4-fucosidase	11.04	10.43
BKKJ1_2071	*fumB*	L-fucose mutarotase	12.80	12.17
BKKJ1_2072	*fumF*	L-2-keto-3-deoxy-fuconate aldolase	13.14	10.86
BKKJ1_2073	*fumD*	L-fuconolactone hydrolase	14.15	8.21
BKKJ1_2074	*fumC*	L-keto-3-deoxy-fuconate-4-dehydrogenase	15.95	15.37
BKKJ1_2075	*fumE*	L-fuconate dehydratase	17.29	13.90
BKKJ1_2076	*fumS*	ABC transporter solute binding protein	19.68	30.20
BKKJ1_2077	*fumT1*	ABC transporter permease	20.79	22.82
BKKJ1_2078	*fumT2*	ABC transporter permease	21.89	30.41
BKKJ1_2079	*fumR*	LacI family transcriptional regulator	—	—

The level of expression is shown as a fold-value of increase in expression on each carbohydrate, as compared to a sorbitol or lactose control, with a cut-off of a minimum 5.0-fold increase in expression. Microarray data were obtained using *B. kashiwanohense* APCKJ1 grown on 1% 2′-FL and were compared with array data obtained when *B. kashiwanohense* APCKJ1 was grown on sorbitol or lactose as a control. The cut-off point was set at 5.0-fold, with a P value of <0.001. With the - sign are indicated fold values below the cut-off. The predicted functions were determined using a combination of BLASTP^71^, Pfam (http://pfam.xfam.org) and KEGG (http://www.genome.jp/kegg/) searches.

Interestingly, when comparing the transcriptomes of a 2′-FL versus lactose-grown APCKJ1 culture, significant transcriptional upregulation was noted of gene BKKJ1_0429 (designated here as *fumG*), predicted to encode 1,2-PD oxidoreductase. 1,2-PD has previously been identified as a product of L-fucose metabolism in certain bifidobacteria^33,39^, and the presence of this gene is consistent with the results of the fermentation product analysis which suggests the utilisation of L-fucose derived from 2′-FL/3-FL by APCKJ1. Between the *lacAS* genes and the *fum* gene cluster we detected an apparently truncated LacI-type repressor-coding gene, *lacR* (Table 1).

Comparison of the transcriptomes of a 2′-FL versus sorbitol grown APCKJ1 culture revealed downregulation of three distinct gene clusters (with locus tags BKKJ1_0067-0069, BKKJ1_0206-0208 and BKKJ1_0336-0341), which, as expected, appear to be involved in sorbitol metabolism based on BLAST analysis (Table 1).

Similar results were obtained when we performed transcriptome analyses for *B. kashiwanohense* APCKJ1 grown on 3-FL (available in the GEO dataset provided here).

### Biochemical characterisation of FumA1 and FumA2, and activity towards 2′-FL and 3-FL

**Figure 3:**
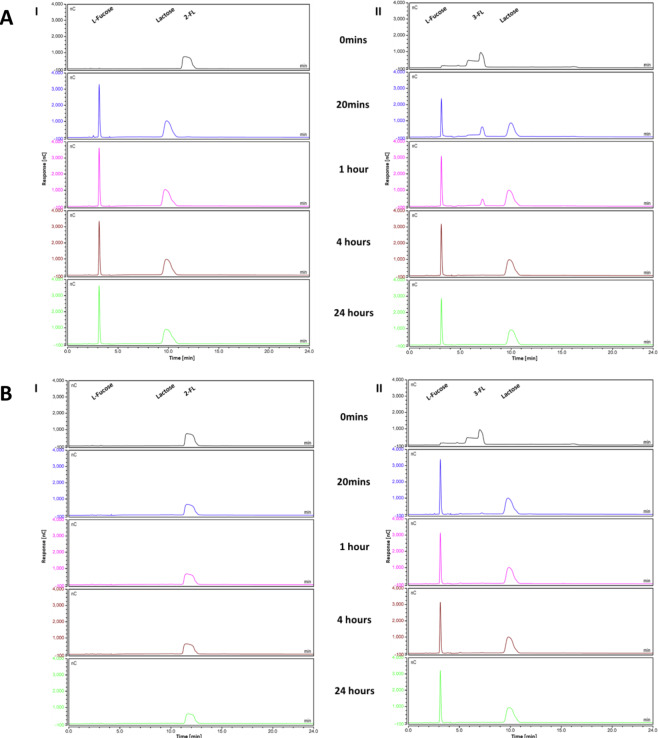
Hydrolysis products of 2′-FL and 3-FL incubated with FumA1His and FumA2His. (**A)** HPAEC-PAD chromatogram profiles of **(I)** 2′-FL and **(II)** 3-FL, when incubated in MOPS buffer (pH7) with FumA1His at the time-points 0 minutes, 20 minutes, 1 hour, 4 hours and 24 hours. (**B)** HPAEC-PAD chromatogram profiles of **(I)** 2′-FL and **(II)** 3-FL, when incubated in MOPS buffer (pH7) with FumA2His at the time-points 0 minutes, 20 minutes, 1 hour, 4 hours and 24 hours. Figure adapted from thesis Figure 5.4; James, 2018^97^.

In order to investigate the predicted fucosidase activities encoded by *fumA1* (BKKJ1_2069) and *fumA2* (BKKJ1_2070), the corresponding FumA1 and FumA2 proteins were purified as His-tagged versions (and designated here as FumA1His and FumA2His, respectively; see Materials and Methods). FumA1 is an 87.46 kDa protein belonging to the GH95 family fucosidases, and FumA2 is a 53.59 KDa protein belonging to the GH29 family fucosidases. Neither protein sequence contains a predicted signal peptide site, indicating their likely intracellular localisation. Assessment of substrate specificity was performed by incubating either FumA1His or FumA2His on their own or in combination with 2′-FL or 3-FL, and analysing the reaction products after varying times (20 minutes, 1 hour, 4 hours and 24 hours) by High-Performance Anion-Exchange Chromatography with Pulsed Amperometric Detection (HPAEC-PAD). Purified FumA1His was shown to fully liberate lactose and L-fucose from both 2′-FL (within 20 minutes) and 3-FL (within 4 hours) (Fig. 3), demonstrating hydrolytic activity towards both the Fuc-α1-2Gal linkage in 2′-FL and the Fuc-α1-3Glc connection in 3-FL, thus indicating a key role in the hydrolysis and utilisation of both sugars. Purified FumA2His was shown to fully cleave 3-FL (within 20 mins), but not 2′-FL, into lactose and L-fucose (Fig. 3), demonstrating hydrolytic activity solely towards the Fumα1-3Glc residue. These results indicate a high hydrolytic activity and specificity of FumA1His for 2′-FL, and similarly of the FumA2His enzyme for 3-FL, with FumA1His also capable of hydrolysing 3-FL, albeit at an apparently low rate.

According to our phylogenetic analysis the FumA1 protein is a as GH95 α-fucosidase based on the fact that it clusters with other known and characterized GH95 members (Fig. S2). Among these are Blon_2335 from *Bifidobacterium longum* subsp. *infantis* ATCC 15697^44^, AfcA from *B. bifidum*^45^ and Afc3 from *Clostridium perfringens*^46^. Of note, GH95 enzymes, also referred as 1,2-α-L-fucosidases (EC 3.2.1.63), specifically target α-1,2-fucosidic linkages^44,46^, thus corroborating our observation of FumA1 showing preference towards 2′-fucosyllactose. In contrast, FumA2 clusters with other previously characterized GH29 α-fucosidases, including BT_2970, BT_2192 and BT3798 from *Bacteroides thetaiotaomicron* VPI-5482^47–49^, Blon_2336 from *Bifidobacterium longum* subsp. *infantis* ATCC 15697^44^, TM0306 from *Thermotoga maritima*^50^ and Afc2 from *Clostridium perfrigens*^46^. Previous studies have demostrated that the GH29 family is further divided into two sub-families with different substrate specificity^24,48^. Notably, FumA2 clusters with previously characterized representatives of GH29 subfamily B (e.g. BT2192 from *B. thetaiotaomicron* and Blon_2336 from *B. longum* subsp. *infantis*) (Fig. S2), indicating that FumA2 is a member of the B subfamily. GH29-B enzymes, also referred as 1,3-1,4-α-l-fucosidases (EC 3.2.1.111), have been shown to be specifically active towards α1,3/4-fucosidic linkages^44,48^, in line with our finding that FumA2 has preference towards 3-fucosyllactose.

### Phenotypic analysis of *B. breve* UCC2003 strains harbouring *B. kashiwanohense* APCKJ1 genes implicated in 2′-FL/3-FL metabolism

**Figure 4:**
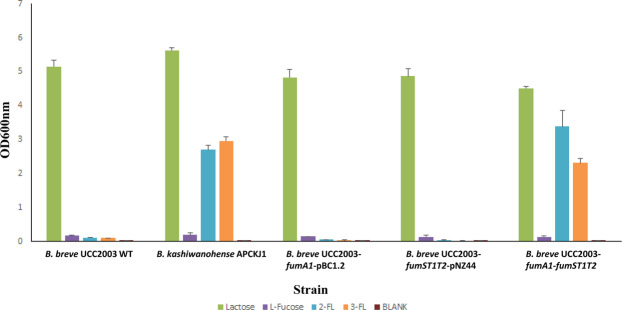
Growth assays of *B. breve* UCC2003 WT, *B. kashiwanohense* APCKJ1 WT and recombinant *B. breve* UCC2003-*fumA1*-*fumST1T2* strains in lactose, L-fucose, 2′-FL or 3-FL. Final OD_600nm_ values after 24 hours of growth of wild type *B. breve* UCC2003, wild type *B. kashiwanohense* APCKJ1 and recombinant *B. breve* UCC2003 strains in modified MRS containing 1% (wt/vol) lactose, L-fucose, 2′-FL or 3-FL as the sole carbon source. The results are the mean values obtained from three separate experiments. Error bars represent the standard deviation. Figure adapted from thesis Figure 5.5; James, 2018^97^.

It has previously been shown that L-fucose supports rather moderate growth of the infant-associated species *B. breve*^18,51^. While some strains of *B. breve* have been demonstrated to metabolize 2′-FL^33,52^, the majority of studied strains, including UCC2003, cannot use 2′-FL or 3-FL as a sole carbon source to any appreciable extent^17,18,33^. Thus, *B. breve* UCC2003 is capable of successfully metabolising the two individual components that make up fucosyllactose (i.e. lactose and L-fucose), yet not the 2′-FL or 3-FL compounds themselves, whereas *B. kashiwanohense* APCKJ1 is capable of growth on 2′-FL, 3-FL and lactose, but not on L-fucose. We therefore elected to heterologously express genes from the *B. kashiwanohense* APCKJ1 *fum* gene cluster, that are predicted to be required for fucosyllactose uptake and hydrolysis in an attempt to confer 2′-FL and/or 3-FL metabolism to *B. breve* UCC2003. The *fumA1* gene was selected because of its ability to hydrolyse both 2′-FL and 3-FL (see above), while the *fumS*, *fumT1* and *fumT2* genes (corresponding to locus tags BKKJ1_2076–2078) were chosen as these genes are predicted to encode the (2′-FL and 3-FL) transporter components of the *fum* locus (Table 1). Accordingly, the *fumA1* gene, and the *fumS*, *fumT1* and *fumT2*-encompassing DNA fragment were cloned into two separate vectors, which were then introduced into *B. breve* UCC2003, generating strain *B. breve* UCC2003-*fumA1*-*fumST1T2* (control strains were constructed containing the ‘empty’ cloning vectors, see Materials and Methods and below). *B. breve* UCC2003-*fumA1*-*fumST1T2* was assessed for its ability to grow in mMRS supplemented with 2′-FL, 3-FL, L-fucose or a lactose control, as compared to the following strains: wild-type UCC2003, *B. breve* UCC2003-*fumA1*-pBC1.2, UCC2003-*fumST1T2*-pNZ44-*strepR*, and wild-type *B. kashiwanohense* APCKJ1 (Fig. 4). Strains *B. breve* UCC2003-*fumA1*-pBC1.2 and UCC2003-*fumST1T2*-pNZ44-*strepR* were generated and used as negative controls, as each of these strains expresses either of the two components predicted as required for 2′-FL/3-FL utilisation *fumA1* and *fumST1T2* (i.e. fucosidase activity and 2′-FL/3-FL transport, respectively), which are both expressed by *B. breve* UCC2003-*fumA1*-*fumST1T2*. Our results demonstrated that wild-type UCC2003, as well as the recombinant UCC2003 strains expressing either *fumA1* or *fumST1T2* on their own, were unable to grow on 2′-FL or 3-FL (final OD_600nm_ < 0.5). In contrast, the recombinant UCC2003 strain expressing both *fumA1* and *fumST1T2* demonstrated good growth on either 2′-FL or 3-FL (final OD_600nm_ > 2.0), which is comparable with that obtained by APCKJ1 grown on either of these substrates (Fig. 4). As expected all of the tested cultures grew to a high cell density on lactose (OD_600nm_ > 3.0), and none of the strains demonstrated significant growth on L-fucose. Notably, this latter result contrasts with previous work which observed the ability of UCC2003 to grow on L-fucose, though at a modest level^51^. However, some L-fucose metabolism by UCC2003 was observed here, based not on growth ability but analysis of metabolic end products, as described below.

Thus, the transcriptome data, substrate hydrolysis profiles and growth results of various recombinant strains corroborate the notion that in *B. kashiwanohense* APCKJ1 the fucosidase encoded by *fumA1* is specifically required for 2′-FL hydrolysis, and capable of hydrolysing 3-FL, while the solute binding protein and two permeases encoded by *fumS*, *fumT1* and *fumT2*, respectively, are responsible for the uptake and internalisation of 2′-FL and 3-FL. These results also demonstrate the ability of a recombinant *B. breve* UCC2003-derivative to successfully internalise and utilise 2′-FL and 3-FL for growth, with the heterologous and concomitant expression of this transport system and fucosidase from *B. kashiwanohense* APCKJ1.

### Analysis of metabolites generated by fucose and fucosyllactose utilisation

NMR spectroscopy of the supernatants generated during growth of *B. kashiwanohense* APCKJ1, *B. breve* UCC2003 or *B. breve* UCC2003-*fumA1*-*fumST1T2* on lactose, L-fucose, 2′-FL or 3-FL, revealed the metabolic end-products of the fermentation of these sugars by these strains. A combination of standard 1D nuclear magnetic resonance spectroscopy (NMR) and 2D Homonuclear ^1^H–^1^H Total Correlation Spectroscopy (TOCSY) and ^1^H J-resolved (JRES) experiments confirmed the presence of a number of metabolites/carbohydrates, including: 1,2-PD, ethanol, lactate, acetate, methanol (contaminant of the 3-FL substrate, see below), formate, lactose, fucose, 2′-FL, 3-FL, α-glucose and β-glucose (Table 2).

**Table 2:** Millimolar (mM) concentrations of metabolites present in the cell-free supernatants of mMRS containing 1% lactose, L-fucose, 2′-FL or 3-FL, following 24 hours incubation with *B. breve* UCC2003 WT, *B. kashiwanohense* APCKJ1 WT, *B. breve* UCC2003 *fumA1*-*fumST1T2*, or with no inoculum (controls). 2′-FL, 2′-fucosyllactose; 3-FL, 3-fucosyllactose; aGluc, α-glucose; ND, not detected.

Culture ID	1,2-propanediol	Ethanol	Lactate	Acetate	Methanol	Formate	L-Fucose	2′-FL	3′-FL	aGluc
No inoculum 1% lactose	ND	ND	3.5 mM	3.8 mM	ND	1.0 mM	ND	ND	ND	1.3 mM
No inoculum 1% fucose	ND	ND	3.8 mM	3.7 mM	ND	1.0 mM	38.4 mM	ND	ND	1.3 mM
No inoculum 1% 2′-FL	ND	ND	3.5 mM	3.7 mM	ND	1.0 mM	ND	20.47 mM	ND	1.3 mM
No inoculum 1% 3-FL	ND	ND	3.5 mM	3.8 mM	1.3 mM	1.0 mM	ND	ND	19.8 mM	1.5 mM
No inoculum mMRS only	ND	ND	3.6 mM	4.0 mM	ND	1.0 mM	ND	ND	ND	1.3 mM
*B. breve* UCC2003 WT 1% lactose	ND	2.0 mM	6.7 mM	87.0 mM	ND	4.0 mM	ND	ND	ND	1.3 mM
*B. breve* UCC2003 WT 1% fucose	20.7 mM	6.7 mM	3.4 mM	25.4 mM	ND	19.7 mM	22.1 mM	ND	ND	ND
*B. breve* UCC2003 WT 1% 2′-FL	ND	1.3 mM	3.4 mM	6.7 mM	ND	1.6 mM	0.6 mM	20.4 mM	ND	1.2 mM
*B. breve* UCC2003 WT 1% 3-FL	ND	trace	3.4 mM	7.5 mM	1.3 mM	1.6 mM	ND	ND	20.5 mM	2.0 mM
*B. kashiwanohense* APCKJ1 WT 1% lactose	ND	1.7 mM	6.6 mM	91.7 mM	ND	3.0 mM	ND	ND	ND	1.5 mM
*B. kashiwanohense* APCKJ1 WT 1% fucose	1.7 mM	2.0 mM	3.3 mM	9.7 mM	ND	5.2 mM	36.1 mM	ND	ND	1.2 mM
*B. kashiwanohense* APCKJ1 WT 1% 2′FL	4.6 mM	3.8 mM	3.4 mM	62.8 mM	ND	9.0 mM	ND	3.3 mM	ND	0.9 mM
*B. kashiwanohense* APCKJ1 WT 1% 3′FL	4.6 mM	2.7 mM	6.0 mM	58.9 mM	1.3 mM	8.0 mM	ND	ND	3.5 mM	1.3 mM
*B. breve* UCC2003 *fumA1-fumST1T2* 1% lactose	ND	7.9 mM	6.0 mM	84.0 mM	ND	2.8 mM	ND	ND	ND	1.3 mM
*B. breve* UCC2003 *fumA1-fumST1T2* 1% fucose	9.8 mM	8.6 mM	3.4 mM	14.5 mM	ND	10.9 mM	38.3 mM	ND	ND	1.2 mM
*B. breve* UCC2003 *fumA1-fumST1T2* 1% 2′-FL	5.1 mM	9.0 mM	5.4 mM	61.6 mM	ND	12.0 mM	10.8 mM	ND	ND	1.3 mM
*B. breve* UCC2003 *fumA1-fumST1T2* 1% 3-FL	7.8 mM	9.9 mM	4.0 mM	68.2 mM	1.3 mM	15.0 mM	7.6 mM	ND	ND	1.3 mM

These identified metabolites/carbohydrates were assigned to peaks in the resulting JRES spectra for each sample, using the TOCSY experiment to identify ^1^H-^1^H coupling in the molecules and allowed accurate identification and quantification of each individual compound within the analyzed samples. The millimolar concentrations of each of these compounds within a given sample, as well as in the medium of the uninoculated controls are represented in Table 2. For all three strains, growth on lactose resulted in a decrease in the amount of lactose present in the samples. This coincided with a substantial increase in the concentration of acetate, and to a lesser extent ethanol, lactate and formate, as compared to the uninoculated control containing 1% lactose. Interestingly, while none of the strains demonstrated significant growth on L-fucose as a substrate, the metabolite profiles of supernatants obtained following incubation of each of the three with this sugar did not match that of the uninoculated control containing 1% L-fucose. In particular, the supernatant of the wild type UCC2003 showed a marked decrease in the concentration of L-fucose present, with an increase in the levels of acetate and formate and 1,2-PD, as well as a small increase in ethanol concentration. The same trend was observed, but to a lesser extent, in the supernatants of UCC2003-*fumA1*-*fumST1T2*, and even less so, APCKJ1, when incubated in the presence of 1% L-fucose. The corresponding metabolite profiles of the supernatants containing 2′-FL and 3-FL were essentially identical, except for the difference between signals originating from the two sugars themselves. The supernatants of wild type UCC2003 incubated in the presence of 2′-FL or 3-FL showed no decrease in the concentration of either sugar, nor the accumulation of additional metabolites, as compared to the uninoculated controls. The supernatants of APCKJ1 grown on 2′-FL or 3-FL revealed a near-complete depletion of either sugar, with significant increases in the concentrations of 1,2-PD, formate, and in particular acetate. The same trend in metabolite profiles was observed for the supernatants of UCC2003-*fumA1*-*fumST1T2* grown on either 2′-FL or 3-FL, with full depletion of these two sugars. These trends in metabolite profiles between the individual samples were further confirmed by PCA analysis, which showed distinct clustering of the *B. kashiwanohense* APCKJ1 WT and the UCC2003-*fumA1-fumST1T2* strains with the exception of the isolates grown on fucose, which clustered independently regardless of bacterial strain (Fig. S3). The presence of a low level of methanol in all 3-FL-containing samples (including the uninoculated control) is presumed to be due to contamination of 3-FL used for these experiments with this alcohol.

### Transcriptome analysis of recombinant *B. breve* UCC2003 grown on 2′-FL

The results of the metabolite analysis indicate that, while neither *B. breve* UCC2003 nor *B. kashiwanohense* APCKJ1 are capable of efficient growth using L-fucose as a sole carbohydrate source, they both still demonstrate the ability to consume a limited quantity of this sugar, and convert it into organic acids and 1,2-PD, in particular (WT) UCC2003. However, the genes involved in this metabolic process in *B. breve* UCC2003 cannot be identified by transcriptomic analysis during this metabolism of free L-fucose, as growth is not sufficient to allow this.

Thus, transcriptomic analyses were carried out on the recombinant *B. breve* strain generated in this study during growth on fucosyllactose, in order to identify the potential genes specifically involved in the metabolism of the L-fucose component of fucosyllactose. Global gene expression was determined by microarray analysis during growth of UCC2003-*fumA1*-*fumST1T2* in mMRS supplemented with 2′-FL, as compared with gene expression during growth in mMRS supplemented with ribose. As lactose is the other constituent of 2′-FL, microarray analysis was also carried out to compare gene expression during growth of UCC2003-*fumA1*-*fumST1T2* in mMRS supplemented with lactose (as compared to gene expression during growth in mMRS supplemented with ribose), in order to identify the lactose metabolism-specific genes expressed during the utilisation of fucosyllactose. Genes that were shown to be significantly upregulated in transcription above the designated cut-off (fold-change > 2.0, P < 0.001) are displayed in Table 3. A fold-change of 2.0 was chosen as the threshold for differential transcription, as this allowed a clear resolution of those genes that exhibited the highest level of increased transcription (from a presumed background of a high number of genes that elicit a lower level of increased transcription).

**Table 3:** *B. breve* UCC2003-*fumA1*-*fumST1T2* genes that were transcriptionally upregulated during growth in mMRS medium supplemented with 2′-FL or lactose as the sole carbohydrate, as compared to growth in mMRS supplemented with ribose.

Gene ID	Gene name	Function	2′-FL	Lactose
Bbr_0526	*lntR*	LacI transcriptional regulator	—	—
Bbr_0527	*lntP1*	ABC transporter permease	2.61	3.81
Bbr_0528	*lntP2*	ABC transporter permease	3.35	2.12
Bbr_0529	*lntA*	GH42 Beta-galactosidase	2.48	2.21
Bbr_0530	*lntS*	ABC transporter solute binding protein	—	—
Bbr_1551	*lacS*	Galactoside symporter	18.72	22.27
Bbr_1552	*lacZ6*	GH2 Beta-galactosidase	12.98	9.05
Bbr_1553	*lacI*	LacI transcriptional regulator	—	—
Bbr_1740	*fumF*	L-2-keto-3-deoxy-fuconate aldolase	4.16	—
Bbr_1741	*fumD*	L-fuconolactone hydrolase	4.69	—
Bbr_1742	*fumP*	L-fucose permease	4.49	—
Bbr_1743	*fumC*	L-keto-3-deoxy-fuconate-4-dehydrogenase	3.70	—
Bbr_1744	*fumE*	L-fuconate dehydratase	7.12	—
Bbr_1745	*fumR*	LacI transcriptional regulator	—	—

The level of expression is shown as a fold-value of increase in expression on each carbohydrate, as compared to a ribose control, with a cut-off of a minimum 2.0-fold increase in expression. Microarray data were obtained using *B. breve* UCC2003-*fumA1*-*fumST1T2* grown on 1% 2′-FL or lactose and were compared with array data obtained when *B. breve* UCC2003-*fumA1*-*fumST1T2* was grown on ribose as a control. The cut-off point was set at 2.0-fold, with a P value of <0.001. With the - sign are indicated fold values below the cut-off. The predicted functions were determined using a combination of BLASTP^71^, Pfam (http://pfam.xfam.org) and KEGG (http://www.genome.jp/kegg/) searches.

Among the genes that showed increased transcription when UCC2003-*fumA1*-*fumST1T2* was grown on lactose or 2′-FL were genes from loci known to function in lactose metabolism, namely the *lnt* (Bbr_0526-0530)^30^ and *lac* loci (Bbr_1551-1553)^53^. Among the genes that were uniquely upregulated in transcription during growth on 2′-FL were those of the gene cluster Bbr_1739-1748 (containing genes, homologs of which are also present in the *fum* locus of APCKJ1) (Table 3 and Fig. S1). These results indicate that while expression of the non-native *B. kashiwanohense* APCKJ1 genes *fumA1*, *fumS*, *fumT1* and *fumT2* in *B. breve* UCC2003 allows the latter strain to take up 2′-FL and to hydrolyse it into lactose and L-fucose, expression of native UCC2003 genes of the *lnt*, *lac* and *fum* loci are presumed to enable strain UCC2003-*fumA1*-*fumST1T2* to further metabolise the released carbohydrates.

### Elucidation of the fucosyllactose/fucose metabolic pathway in *B. kashiwanohense* and *B. breve*

The results of the APCKJ1 transcriptome analyses indicate involvement of the *fum* locus and *fumG* in fucosyllactose metabolism, and the combined results of the *in vitro* hydrolytic assays and recombinant UCC2003 growth analysis implicate the APCKJ1 fucosidase-encoding genes *fumA1* and *fumA2*, as well as the transporter-encoding genes *fumS*, *fumT1* and *fumT2* in the internalisation and hydrolysis of 2′-FL and 3-FL. However, the presence of further genes of the *fum* locus, as well as *fumG*, upregulated in transcription during growth on 2′-FL or 3-FL suggests the involvement of these genes in the metabolism of the liberated L-fucose, given that the genes for lactose metabolism appear to be constitutively expressed in strain APCKJ1. Likewise, transcriptional upregulation of genes in the UCC2003 *fum* locus during growth of the recombinant strain on 2′-FL implicate these genes in L-fucose metabolism in UCC2003. This, and the NMR data, suggests that both *B. kashiwanohense* APCKJ1 and *B. breve* UCC2003 possess functional pathways for the metabolism of L-fucose.

A pathway for the anaerobic catabolism of L-fucose leading to the formation of 1,2-PD (via non-phosphorylated intermediates) has recently been proposed for *B. longum* subsp. *infantis* ATCC15697^33^, based on sequence homology with enzymes from a fucose-dependent metabolic route previously identified in *Xanthomonas campestris*^54^. Genes involved in the proposed L-fucose utilisation pathway in *B. longum* subsp. *infantis* ATCC15697 were used in BLASTP searches for homologs in the genomes of *B. kashiwanohense* APCKJ1 and *B. breve* UCC2003. Homologs identified in the APCKJ1 and UCC2003 genomes, whose predicted annotations did not match the annotations of their ATCC15697 equivalents (based on their function in the fucose/fucosyllactose utilisation pathways^33^), were further checked for alternative annotations in the Pfam (http://pfam.xfam.org) and KEGG (http://www.genome.jp/kegg/) databases.

**Figure 5:**
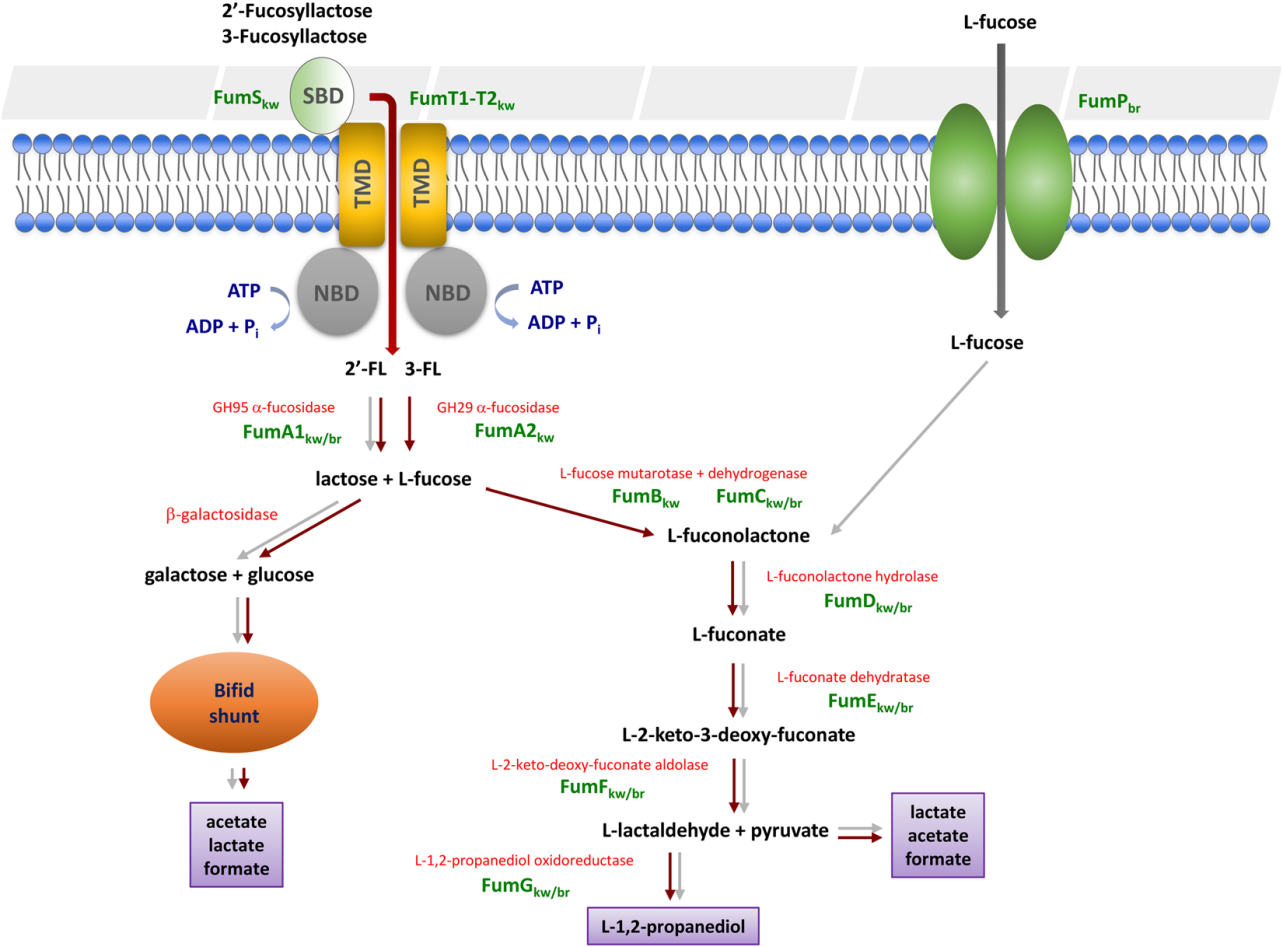
Fucose utilisation pathway in *B. kashiwanohense* and *B. breve*. Schematic representation of the pathway for the utilisation of fucosyllactose in *B. kashiwanohense* APCKJ1 (red arrows) and *B. breve* UCC2003 (grey arrows). Figure adapted from thesis Figure 5.6; James, 2018^97^.

This search revealed the presence of homologs for all predicted fucose utilisation genes from ATCC15697 in APCKJ1 (Table S2), including seven of the genes of the *fum* locus, and the gene *fumG*, all of which were transcriptionally upregulated when APCKJ1 was grown on 2′-FL (see above). These results therefore suggest the presence of all genes necessary for L-fucose metabolism in *B. kashiwanohense* APCKJ1, via a pathway converting L-fucose into L-2-keto-3-deoxyfuconate (as proposed in *B. breve*, *B. longum* subsp. *infantis*, and *B. longum* subsp. *suis*^33,39^), but subsequently converted into L-lactaldehyde and pyruvate by means of a predicted L-2-keto-deoxy-fuconate aldolase (FumF) encoded by the *fum* locus (Fig. 5). The presence of an L-1,2-propanediol oxidoreductase (FumG) in the genome sequence of APCKJ1 allows the further reduction of L-lactaldehyde into L-1,2-PD (Fig. 5). These findings are supported by the observed accumulation of lactate, acetate, formate (all metabolites of pyruvate) and 1,2-PD in the 2′-FL and 3-FL fermentates analysed by HPLC and NMR, as mentioned above.

Homologs for the *fum* genes in APCKJ1 were identified in the UCC2003 genome (Table S2) and were shown to be transcriptionally upregulated when the recombinant *B. breve* UCC2003 was grown on 2′-FL (Table 3). Notably, a recent transcriptional study conducted on *B. breve* UCC2003 identified the FumG homologue (Bbr_1505) as constitutively expressed in this strain^55^.

**Figure 6:**
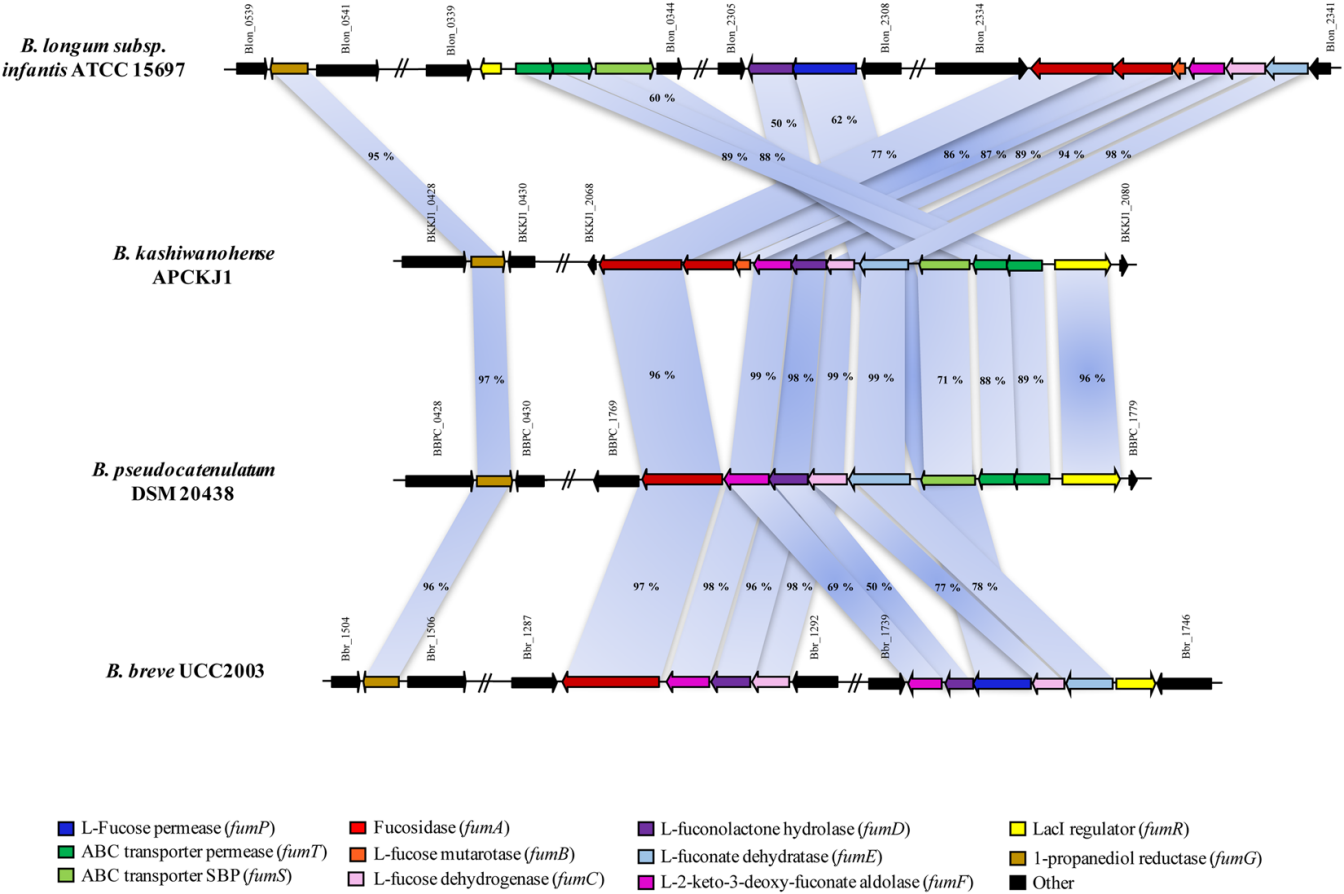
Fucose utilisation loci in infant-derived bifidobacterial strains. Comparison of the fucose/fucosyllactose metabolism genes of *B. kashiwanohense* APCKJ1 with corresponding homologous loci of *B. longum* subsp. *infantis* ATCC 15697, *B. pseudocatenulatum* DSM 20438 and *B. breve* UCC2003, as identified using the heatmap in Fig. 7. Each solid arrow represents an open reading frame. The length of each arrow is proportional to the size of the open reading frame, and the color code is indicative of the predicted gene function. The amino acid sequence identity of each predicted protein expressed as a percentage is also shown.

The ATCC15697 genes lacking homologs in UCC2003 were the GH29 α-fucosidase-encoding gene and the L-fucose mutarotase-encoding gene, as well as those encoding the components the (three) components of the proposed fucosyllactose uptake system. While neither *B. breve* UCC2003 nor *B. kashiwanohense* APCKJ1 demonstrated significant growth on free L-fucose, our NMR data indicate active metabolism of this sugar in both species, generating the same metabolites (although at lower levels) as are observed following growth on 2′-FL or 3-FL. Thus, the apparent absence of an L-fucose mutarotase does not seem to prevent metabolism of L-fucose in UCC2003. While a GH95 fucosidase-encoding gene homologous to *fumA1*_*kw*_ (Bbr_1288) was identified within an additional “HMO islet” in UCC2003 (Bbr_1288-91) (Fig. 6), the presence of 2′-FL or 3-FL do not seem to induce transcription of this region, as demonstrated in the recombinant UCC2003 *fumA1-ST1T2* growth assays. It is possible that this locus containing *fumA1*_*br*_ targets other fucosylated HMO structures, such as fucosyl-LN(n)T, as *B. breve* is known to efficiently consume free LN(n)T^30^. Alternatively, it may be that this is a non-functional remnant of a former FL-utilization gene cluster.

### Distribution of fucose/fucosyllactose utilisation genes in the *Bifidobacterium* genus

The results of the microarray analysis implicated the protein products of *fumG* (BKKJ1_0429) as well as the genes of the *fum* locus (BKKJ1_2069-2079) in the utilisation of fucosyllactose by *B. kashiwanohense* APCKJ1. This is supported by the results of the phenotypic analysis of the *B. breve* UCC2003 strain expressing the fucosidase FumA1 and associated transporter proteins encoded by this locus. We selected four genes within the *fum* locus responsible for the intracellular metabolism of fucosyllactose and/or L-fucose identified in APCKJ1 in order to observe their distribution within the *Bifidobacterium* genus. The regulatory and transport-associated genes of the *fum* locus were excluded from comparative analysis, as carbohydrate transporter and transcriptional regulation-associated genes generally share a high degree of homology across bifidobacteria, thus lacking distinguishable sequence variability^56,57^.

**Figure 7:**
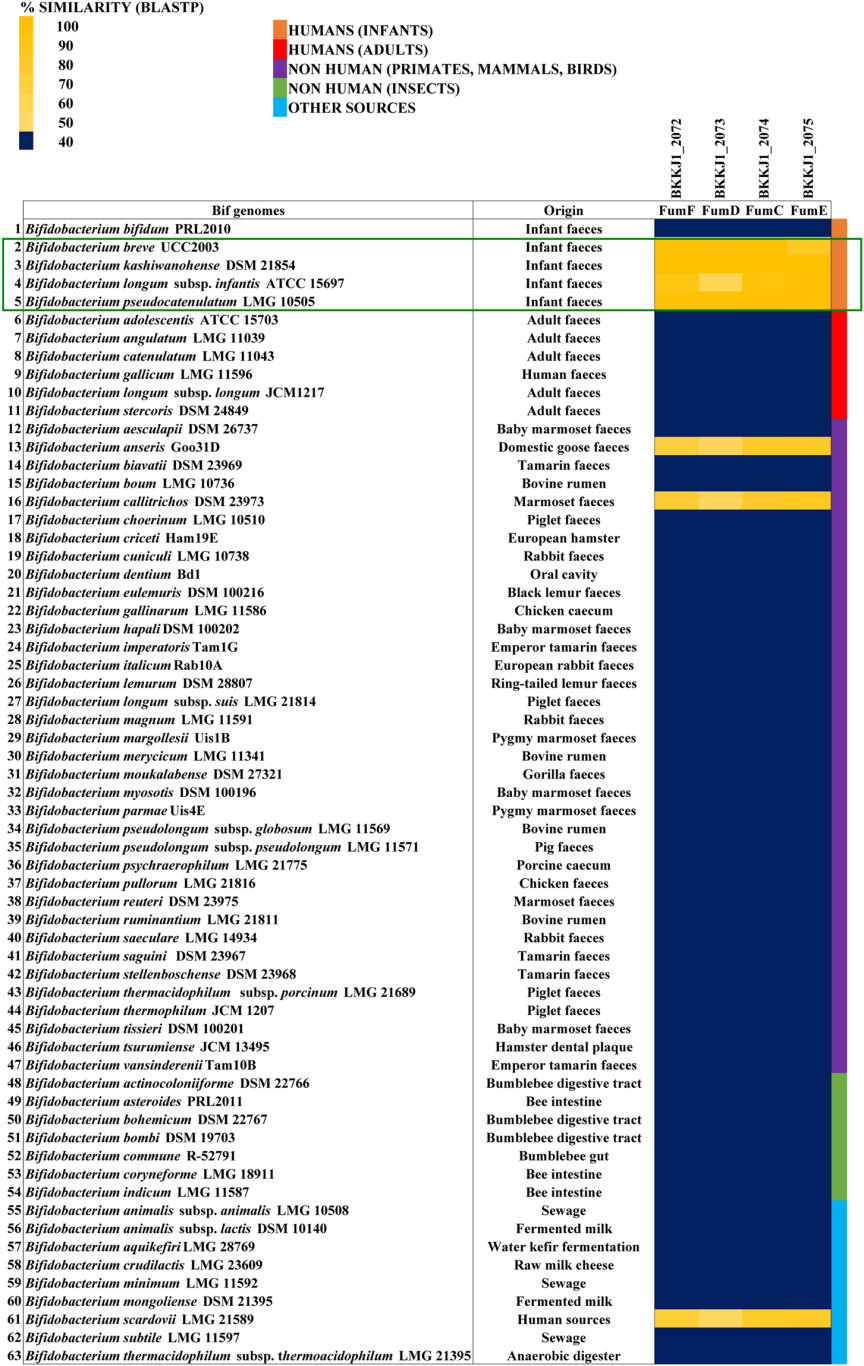
Distribution of *fum* genes across the *Bifidobacterium* genus. Heatmap representing the distribution of homologs of four genes encoding proteins predicted to have a key role in fucose/fucosyllactose utilisation pathway from *B. kashiwanohense* APCKJ1 across the *Bifidobacterium* genus. Gene products from the representative type strain genomes of all online-available *Bifidobacterium* species with a significant homology of 50% iterative similarity over 50% of protein length are represented in the matrix. The colour code grading represents the degree of sequence similarity at protein level, with the species grouped by origin of isolation.

This analysis revealed that the four genes included in the analysis are predominant across infant-associated bifidobacteria (e.g. *B. kashiwanohense, B. breve*, *B. longum* subsp. *infantis* and *B. pseudocatenulatum*), with the exception of *B. bifidum*. (Fig. 7). This corroborates the observation that members of the species *B. bifidum* employ extracellular fucosidases to liberate the lactose component of 2′-FL and 3-FL, which is subsequently metabolised, but are not capable of metabolising the released L-fucose^58^. The degree of similarity between the fucose utilisation genes from *B. kashiwanohense* APCKJ1 and their corresponding homologous loci in *B. longum* subsp. *infantis* ATCC15697 and *B. breve* UCC2003 are shown in Table S2.

## Discussion

*B. kashiwanohense* represents an example of an apparently infant-specific *Bifidobacterium* species, although just two publications have previously reported on the isolation of this species from infant faeces^34,35^. The bifidobacterial component of the infant gut microbiota is typically dominated by the species *B. breve*, *B. longum* subsp. *longum* and *B. bifidum*, with *B. pseudocatenulatum*, *B. longum* subsp. *infantis* and *B. longum* subsp. *suis* occurring at lower frequency^13,59–61^. However, the ability of members of *B. kashiwanohense* to utilise milk-derived substrates suggests that the infant gut may constitute its primary ecological niche. The ability of a *B. kashiwanohense* isolate to grow on the HMOs 2′-FL or 3-FL as its sole carbohydrate source has been reported^33^. HMO utilisation by certain members of the *Bifidobacterium* genus appears to be present in some infant-associated species^8,18,19,29,33^. As fucosylated sugars typically represent a substantial proportion of all HMOs^2,36,37^, the ability to utilise fucosyllactose by *B. kashiwanohense* represents a specific adaptation to the neonatal gut environment. Here, a novel *B. kashiwanohense* strain (APCKJ1) isolated from the faeces of a breastfed infant demonstrated the ability to consume 2′-FL or 3-FL as its sole carbohydrate source, which became the focus of this investigation.

Transcriptional analysis of APCKJ1 revealed an 18 kb gene cluster dedicated to the metabolism of fucosyllactose. This region contains the adjacent *lac* and *fum* loci, thus representing a potential ‘HMO island’ conferring the ability to utilize fucosyllated HMOs. Interestingly, the constitutive transcription of the *lac* locus (Table 1) is due to the presence of a truncated, non-functional LacI-type repressor (LacR) in APCKJ1 strain. Furthermore, homologs of *lacR* appear to be entirely absent in *B. kashiwanohense* DSM21854 and *B. longum* subsp. *infantis* ATCC15697^33^. A homolog of this ‘HMO island’ has been previously described in *B. longum* subsp. *infantis*^26,62,63^ and *B. longum* subsp. *longum*^64^, thus reinforcing the notion of specific adaptations to HMO utilisation by infant-associated bifidobacteria.

The combined results of *in vitro* hydrolysis assays, heterologous expression of FumA1 and FumA2 fucosidases in *B. breve* UCC2003, as well as growth results obtained with recombinant UCC2003 strains confirmed the predicted functions of some of the genes of the *fum* locus. Our findings are consistent with the notion that the three adjacent genes *fumS*, *fumT1* and *fumT2* encode transport components necessary for 2′-FL/3-FL uptake, while the *fumA1* and *fumA2* encoded fucosidases are responsible for the hydrolysis of 2′-FL and 3-FL, respectively. Of note, our findings corroborate what had previously been observed in a subset of FL-utilising bifidobacteria (members of *B. breve*, *B. longum* and *B. pseudocatenulatum* spp), where a solute binding protein of an ABC transporter system and an α-fucosidase (GH95 family) were shown to play an essential role in FL utilisation^38^. Interestingly, while FumA1 is capable of hydrolysing both 2′-FL and 3-FL, it possesses higher catalytic efficiency with the former; FumA2 instead can only hydrolyse 3-FL. Such apparent redundancy in the degradation of isomeric HMO structures is not unusual in bifidobacteria^19,27,30^. However, it is interesting that both FumA1 and FumA2 can hydrolyse 3-FL, as this HMO has been reported to be much less abundant in breastmilk compared to its isomer 2′-FL^36^. We can’t exclude that the true target substrate for FumA2 may be in fact another fucosylated HMO, such as fucosyl-LN(n)T, containing similar linkages.

While these results reveal the mechanisms of fucosyllactose uptake and intracellular hydrolysis in *B. kashiwanohense* APCKJ1, they do not reveal whether small fractions of L-fucose may be internalised and used by the cell. The combined results of the HPLC and NMR analyses of the various 2′-FL/3-FL and L-fucose fermentations, as well at the APCKJ1 microarrays, indicate the utilisation of the L-fucose mainly resulting by the hydrolysis of 2′-FL and 3-FL, with even some partial metabolism of free L-fucose. The comparative analysis performed between *B. kashiwanohense* APCKJ1 and *B. longum* subsp. *infantis* ATCC15697 revealed that all the presumed genetic components necessary for the utilisation of L-fucose are encoded by APCKJ1. This therefore represents the first evidence of a complete and functional fucose utilisation pathway in *B. kashiwanohense*, a species which has been previously suggested to extracellularly release the fucose component of fucosyllactose^33,39^. In addition to this we also identified an identical (predicted) pathway for L-fucose metabolism in *B. breve* UCC2003, as this species has recently been characterised for its utilisation of L-fucose^39^. The observed depletion of L-fucose and accumulation of its breakdown product 1,2-PD show that *B. breve* UCC2003 and *B. kashiwanohense* APCKJ1 possess the ability to at least partially metabolise free L-fucose, despite not growing to any significant degree in its sole presence. Interestingly, 1,2-PD excretion in human urine and faeces has been largely attributed to contamination since it is an excipient in several pharmaceutical preparations or food additives^65^. However, other research has shown that 1,2-PD is present in higher concentrations in faeces of breastfed babies^66^, which would be consistent with microbial influence.

Identification of homologs involved in fucose metabolism across the *Bifidobacterium* genus highlights the apparent importance of this pathway for colonisation of the infant gut. While the ability to utilise fucosyllactose undoubtedly provides an advantage for *B. kashiwanohense* establishment in the breastfed neonatal gut, this strategy is also employed by other infant-associated bifidobacteria, including *B. bifidum*, *B. longum* subsp. *infantis*, *B. longum* subsp. *longum* (and possibly *B. pseudocatenulatum*) as well as some species of *B. breve*, all of which are capable of utilising other HMO components, such as sialyllactose, LN(n)T, LNB, sialic acid and/or L-fucose^8,19–32,64^. Therefore, the narrow specialisation in HMO metabolism by *B. kashiwanohense* may in fact also be a hindrance in becoming one of the dominant species in the breastfed infant gut microbiota.

The findings of this study reinforce the importance of HMO metabolism for the successful proliferation of *Bifidobacterium* species in the breastfed infant gut. This allows the depiction of the metabolic pathway required for the utilisation of fucosyllactose by *B. kashiwanohense* APCKJ1 and a potential pathway for the metabolism of free L-fucose by *B. breve* UCC2003, and furthermore reveals the distribution of fucose-utilisation associated genes in *Bifidobacterium* species typically found in neonates. Our findings not only demonstrate the conservation of fucose metabolism in many infant-associated bifidobacteria, but also highlight the role of fucosylated HMOs in bifidobacterial establishment in the breastfed infant gut.

Thus, the outcome of our work significantly contributes to increasing our understanding of HMO metabolism by infant-associated bifidobacteria. In particular *B. kashiwanohense*, while previously shown to consume HMO^33^, remained until now uncharacterised for its manner of HMO utilisation. Our findings have generated insights into the pathway employed by a number of *Bifidobacterium* species for their metabolism of L-fucose freely available or liberated by the degradation of larger HMO structures. Ultimately, our gained understanding of HMO metabolism by a number of *Bifidobacterium* species can be used as a tool to identify infant-specific probiotic strains and conversely reinforces the potential of fucosylated HMOs as a prebiotic.

## Materials and Methods

### Strain isolation from infant faeces, and species identification

Pure cultures of *Bifidobacterium* strains, capable of utilising 2′-FL as their sole carbohydrate source, were isolated from the faeces of an exclusively breastfed neonate (aged 4 weeks). The faecal sample was obtained from the APC Microbiome Ireland human clinical sample collection (stored at −80 °C), originally collected for the InfantMet Cohort^67^. Mothers were approached for consent between February 2012 and May 2014 at the Cork University Maternity Hospital. All mothers provided informed consent for the use of the infant faeces in this study. Ethical approval provided by the Cork University Hospital Research Ethics Committee (ethical approval reference: ECM (w) 07/02/2012), with all methods performed in accordance with the relevant guidelines and regulations required by said committee. *Bifidobacterium* culture isolates were obtained using a method based on that employed in the InfantMet study^67^, though with the following modifications. All sample preparation and platings were carried out under anaerobic conditions in a modular atmosphere-controlled system (Davidson and Hardy, Belfast, Ireland) at 37 °C. One gram of frozen faecal sample was thawed anaerobically at 37 °C, and resuspended in 10 ml PBS (Sigma Aldrich, Ireland) supplemented with 0.05% L-cysteine hydrochloride (Sigma Aldrich, Ireland). Selection of bifidobacteria was performed by spread-plating 10 aliquots of 1 ml of the faecal resuspension on: modified de Man Rogosa and Sharpe (mMRS) agar^68^, supplemented with 1% (wt/vol) 2′-FL (Glycom A/S, Lyngby, Denmark), 0.05% L-cysteine-HCl, 100 μg/ml mupirocin (Oxoid, Fannin, Ireland) and 50 units nystatin suspension (Sigma Aldrich, Ireland). Agar plates were incubated anaerobically at 37 °C for 72 h. Emerging colonies from these plates were resuspended in 1 ml of PBS supplemented with 0.05% L-cysteine-HCl, after which the resuspensions from the 10 plates of a single sample were pooled and homogenised, and serial-diluted in the aforementioned PBS, and dilutions between 1 × 10^−4^ and 1 × 10^−8^ were spread-plated on 2′-FL-supplemented mMRS agar, as described above, and incubated anaerobically at 37 °C for 72 h. Single, isolated colonies were selected from the serial dilution plates, and re-streaked onto the same agar medium, and incubated anaerobically at 37 °C for 48 h. This sub-cultivation process was repeated twice, after which individual colonies were inoculated into modified mMRS medium, supplemented with 1% lactose and 0.05% L-cysteine-HCl. Pure cultures were stocked and stored at −80 °C. Isolates were confirmed as bifidobacteria based on the fructose-6-phosphate phosphoketolase activity test^69^, and species identity was determined by sequencing of their ITS region^70^.

### Genome sequencing and annotation of novel isolate *Bifidobacterium kashiwanohense* APCKJ1

Genome sequencing of the *B. kashiwanohense* isolate was performed by GATC Biotech Ltd. (Germany) using Pacific Biosciences SMRT RSII technology. Raw sequencing reads were *de novo* assembled using the Hierarchical Genome Assembly Process (HGAP) protocol RS_Assembly.2 implemented in the SMRT Smart Analysis portal v.2.3 with default parameters (https://github.com/PacificBiosciences/SMRT-Analysis). Open Reading Frame (ORF) prediction and automatic annotation was performed using Prodigal v2.0 (http://prodigal.ornl.gov) for gene predictions, BLASTP v2.2.26 (cut-off e-value of 0.0001)^71^ for sequence alignments against a combined bifidobacterial genome-based database, and MySQL relational database to assign annotations. Predicted functional assignments were manually revised and edited using similarity searches against the non-redundant protein database curated by the National Centre for Biotechnology Information (ftp://ftp.ncbi.nih.gov/BLAST/db/) and PFAM database (http://pfam.sanger.ac.uk), allowing a more detailed, *in silico* characterization of hypothetical proteins. GenBank editing and manual inspection was performed using Artemis v18 (http://www.sanger.ac.uk/resources/soft-ware/artemis/). Transfer RNA genes were identified employing tRNAscan-SE v1.4 and ribosomal RNA genes were detected based on Rnammer v1.2^72^ software supported by BLASTN v2.2.26.

### Nucleotide sequence analysis and multiple sequence alignment

Sequence data were obtained from Artemis-mediated^73^ genome annotations of *B. kashiwanohense* strains APCKJ1, JCM15439^74^ and PV20-2^75^, and *B. breve* UCC2003^76^. Database searches were performed using non-redundant sequences accessible at the National Centre for Biotechnology Information (http://www.ncbi.nlm.nih.gov) using BLAST^71^. Sequences were verified and analysed using SeqMan and SeqBuilder programs of the DNAStar software (version 10.1.2; DNAStar, Madison, WI, USA). Multiple sequence alignments of amino acid sequences were created with Clustal Omega (https://www.ebi.ac.uk/Tools/msa/clustalw/), using default settings^77^. Alignments were visualised with Genedoc v 2.7.0 (http://iubio.bio.indiana.edu/soft/molbio/ibmpc/), using physicochemical display mode (similar colouring based on common physical and chemical properties).

### Bacterial strains, plasmids, culture conditions and *Bifidobacterium* growth assays

Bacterial strains and plasmids used in this study are listed in Table S3. *B. breve* and *B. kashiwanohense* cultures were incubated under anaerobic conditions in a modular atmosphere-controlled system (Davidson and Hardy, Belfast, Ireland) at 37 °C. *B. breve* UCC2003 was routinely cultured in either de Man Rogosa and Sharpe medium (MRS medium; Difco, BD, Le Pont de Claix, France) supplemented with 0.05% L-cysteine-HCl and 1% lactose, or reinforced clostridial medium (RCM; Oxoid Ltd., Basingstoke, England). *B. kashiwanohense* APCKJ1 was routinely cultured in modified mMRS supplemented with 0.05% L-cysteine-HCl and 1% lactose. *Lactococcus lactis* strains were cultivated in M17 broth (Oxoid Ltd., Basingstoke, England) containing 0.5% glucose^78^ at 30 °C. *Escherichia coli* strains were cultured in Luria-Bertani (LB) broth^79^ at 37 °C with agitation. Where appropriate, growth media contained chloramphenicol (Cm; 5 μg ml^−1^ for *L. lactis* and *E. coli*, 2.5 μg ml^−1^ for *B. breve*) or streptomycin (Strep; 400 μg ml^−1^).

Carbohydrate utilisation by bifidobacterial strains was examined in mMRS medium^68^, and excluding a carbohydrate source. Prior to inoculation, the mMRS medium was supplemented with cysteine-HCl (0.05%, wt/vol) and a particular carbohydrate source (1%, wt/vol). It has previously been shown that mMRS does not support growth of *B. breve* UCC2003 in the absence of an added carbohydrate^68^. Carbohydrates used were lactose, glucose, D-ribose, sorbitol, cellobiose, raffinose, melibiose (all purchased from Sigma Aldrich, Steinheim, Germany), and LNT, LNnT, lactosamine-hydrochloride (lactosamine HCl), N-acetylneuramic acid (sialic acid), L-fucose, 3-sialyllactose (3-SL), 6-sialyllactose (6-SL), 2′-FL and 3-FL (all obtained from Glycom, Lyngby, Denmark). A 1% wt/vol carbohydrate concentration was considered sufficient to analyse growth capabilities of a strain on a particular carbon source. To determine bacterial growth profiles and final optical densities, 5 ml of freshly prepared mMRS medium, including a particular carbohydrate (see above), was inoculated with 50 μl (1%) of a stationary phase culture of *B. breve* UCC2003 or *B. kashiwanohense* APCKJ1. Uninoculated mMRS medium was used as a negative control. Cultures were incubated anaerobically at 37 °C for 24 h, and the optical density at 600 nm (OD_600nm_) was determined manually. Growth assays were repeated in duplicate or triplicate, depending on carbohydrate availability.

### HPLC analysis

Fermentation end-product profiles were assessed by HPLC following growth of selected bifidobacteria in mMRS medium containing 1% 2′-FL, 3-FL or lactose as the sole carbohydrate source. Collected culture samples were then prepared for HPLC analysis by centrifugation at 3270 × *g* for 5 min, after which the resulting supernatants were filter sterilized (0.45 μm filter, Costar Spin-X Column) and stored at −20 °C until further use. An Agilent 1200 HPLC system (Agilent Technologies, Santa Clara, CA) with a refractive index detector was used to detect generated metabolites. Metabolite peaks were identified based on retention times of known standards (2′-FL, 3-FL, L-fucose, lactose, lactic acid, acetic acid, formic acid, sodium pyruvate and 1,2-PD) at a concentration of 10 mM. Non-fermented mMRS medium containing carbohydrates (or the absence of any carbohydrate) served as controls. A REZEX 8 μm 8% H organic acid column (300 mm × 7.8 mm, Phenomenex, Torrance, CA, USA) was utilized and maintained at 65 °C. Elution was performed for 25 min using a 0.01 M H_2_SO_4_ solution at a constant flow rate of 0.6 mL/min.

### Analysis of global gene expression using *B. breve* DNA microarrays

Global gene transcription was determined during log-phase growth of *B. kashiwanohense* APCKJ1 in mMRS supplemented with 2′-FL. The obtained transcriptome was compared to that determined for log-phase *B. kashiwanohense* APCKJ1 cells when grown in mMRS supplemented with sorbitol. The transcriptome for growth of APCKJ1 on 2′-FL, as compared to a lactose control, was also obtained by the same method. Likewise, global gene transcription was determined during log-phase growth of recombinant strain *B. breve* UCC2003-*fumA1fumST1T2* in mMRS supplemented with either 2′-FL or lactose. The obtained transcriptome was compared to that determined for log-phase *B. breve* UCC2003-*fumA1fumST1T2* cells when grown in mMRS supplemented with ribose.

Microarrays containing oligonucleotides representing each of the 1864 identified open reading frames (ORFs) on the genome of *B. breve* UCC2003, or each of the 2110 identified ORFs on the genome of *B. kashiwanohense* APCKJ1, were designed and obtained from Agilent Technologies (Palo Alto, Ca., USA). Methods for cell disruption, RNA isolation, RNA quality control, complementary DNA synthesis and labelling were performed as described previously^80^. Labelled cDNA was hybridized using the Agilent Gene Expression hybridization kit (part number 5188-5242) as described in the Agilent Two-Colour Microarray-Based Gene Expression Analysis v4.0 manual (publication number G4140-90050). Following hybridization, microarrays were washed in accordance with Agilent’s standard procedures and scanned using an Agilent DNA microarray scanner (model G2565A). Generated scans were converted to data files with Agilent’s Feature Extraction software (Version 9.5). DNA-microarray data were processed as previously described^81–83^. Differential expression tests were performed with the Cyber-T implementation of a variant of the student t-test^84^.

### DNA Manipulations

Chromosomal DNA was isolated from *B. breve* UCC2003 and *B. kashiwanohense* APCKJ1 as previously described^85^. Plasmid DNA was isolated from *E. coli*, *L. lactis* and *B. breve* using the Roche High Pure Plasmid Isolation kit (Roche Diagnostics, Basel, Switzerland). An initial lysis step was performed using 30 mg ml^−1^ of lysozyme for 30 minutes at 37 °C prior to plasmid isolation from *L. lactis, B. breve* or *B. kashiwanohense*. DNA manipulations were essentially performed as described previously^79^. Restriction enzymes and T4 DNA ligase were used according to the supplier’s instructions (Roche Diagnostics, Basel, Switzerland). Synthetic single stranded oligonucleotide primers used in this study (Table S4) were synthesized by Eurofins (Ebersberg, Germany). Standard PCRs were performed using Extensor Hi-Fidelity PCR Master Mix (Thermo Scientific, Waltham, United States) or Q5 High-Fidelity 2X Mastermix (New England BioLabs, Herefordshire, United Kingdom) in a Life Technologies Pro-FLex PCR System (Thermo Scientific, Waltham, United States). PCR products were visualized by ethidium bromide staining following agarose gel electrophoresis (1% agarose). *B. breve* colony PCR reactions were performed as described previously^86^. PCR fragments were purified using the Roche high Pure PCR purification kit, while plasmid DNA was isolated using the Roche High Pure Plasmid Isolation kit (Roche Diagnostics, Basel, Switzerland). Plasmid DNA was introduced into *E. coli* by electroporation as described previously^79^. *B. breve* UCC2003^76^ and *L. lactis*^87^ were transformed by electroporation according to published protocols. The correct fragment orientation and integrity of all plasmid constructs (see also below) were verified by DNA sequencing, performed at Eurofins (Ebersberg, Germany).

### Construction of overexpression vectors, protein overproduction and purification

For the construction of plasmids pNZ-FucA1 and pNZ-FucA2, DNA fragments encompassing the predicted fucosidase-encoding genes *fumA1* (corresponding to BKKJ1_2069) or *fum2* (corresponding to BKKJ1_2070) were generated by PCR amplification from chromosomal DNA of *B. kashiwanohense* APCKJ1 using Q5 High-Fidelity DNA polymerase and the primer combinations 2069F and 2069R, or 2070F and 2070R, respectively (Table S4). An in-frame N-terminal His10-encoding sequence was incorporated into the forward primers 2069F and 2070F to facilitate downstream protein purification. The generated amplicons were digested with SmaI and XbaI, and ligated into the ScaI and XbaI-digested, nisin-inducible translational fusion plasmid pNZ8150^88^. The ligation mixtures were introduced into *L. lactis* NZ9000 by electrotransformation and transformants were then selected based on chloramphenicol resistance. The plasmid content of a number of Cm-resistant transformants was screened by restriction analysis and the integrity of positively identified clones was verified by sequencing.

Nisin-inducible gene expression and protein overproduction was performed as described previously^89–91^. In brief, 400 ml of M17 broth supplemented with 0.5% (wt/vol) glucose was inoculated with a 2% inoculum of a particular *L. lactis* strain, followed by incubation at 30 °C until an OD_600nm_ of 0.5 was reached, at which point protein expression was induced by addition of cell-free supernatant of a nisin-producing strain^92^, followed by continued incubation for a further 2 hours. Cells were harvested by centrifugation and protein purification achieved as described previously^90^. Protein samples were dialysed into 20 mM morpholinepropanesulfonic acid (MOPS) (pH 7.0) buffer using Merck Amicon Ultra-15 Centrifugal Filter Units and stored at 4 °C. Protein concentrations were determined using the Bradford method^93^.

### Assay of individual and combined fucosidase activities

The individual or sequential hydrolytic activities specified by FumA1 and FumA2 were determined essentially as described previously^89^, using either 2′-FL or 3-FL as a substrate. Briefly, a 50 μl volume of each purified protein (protein concentration of 0.5 mg/ml) was added to 20 mM MOPS (pH 7.0) buffer and 1 mg ml^−1^ (wt/vol) of one of the above-mentioned sugars in a final volume of 1 ml, followed by incubation for 24 hours at 37 °C. In order to determine enzyme affinity, each enzyme was incubated individually or in combination, with 2′-FL or 3-FL, as described above, with 200 μl samples taken at 20 minutes, 1 hour, 4 hours and 24 hours. All samples were subject to a final enzyme denaturation step at 85 °C for 15 minutes, before storage at −20 °C.

### HPAEC-PAD analysis

For HPAEC-PAD analysis, a Dionex (Sunnyvale, CA) ICS-3000 system was used. Carbohydrate fractions from the above-mentioned hydrolysis assays (25 μl aliquots) were separated on a CarboPac PA1 analytical-exchange column (dimensions, 250 mm by 4 mm) with a CarboPac PA1 guard column (dimensions, 50 mm by 4 mm) and a pulsed electrochemical detector (ED40) in PAD mode (Dionex). Elution was performed at a constant flow-rate of 1.0 ml/min at 30 °C using the following eluents for the analysis: eluent A, 200 mM NaOH; eluent B, 100 mM NaOH plus 550 mM Na acetate; eluent C, Milli-Q water. The following linear gradient of sodium acetate was used with 100 mM NaOH: from 0 to 50 min, 0 mM; from 50 to 51 min, 16 mM; from 51 to 56 min, 100 mM; from 56 to 61 min, 0 mM. Chromatographic profiles of standard carbohydrates were used for comparison of the results of their breakdown by FumA1 or FumA2 proteins. Chromeleon software (version 6.70; Dionex Corporation) was used for the integration and evaluation of the chromatograms obtained. A 1 mg/ml stock solution of each of the carbohydrates, as well as their putative breakdown products (where available) used as reference standards was prepared by dissolving the particular sugar in Milli-Q water. Chromatographic profiles of mMRS containing standard carbohydrates were used for comparison of the results of their fermentation by *B. kashiwanohense* APCKJ1. mMRS containing 1% of 2′-FL or 3-FL, as well as their putative breakdown products (where available) used as reference standards.

### Generation of recombinant *B. breve* strains

DNA fragments encompassing *fumA1* (corresponding to locus tag BKKJ1_2069); and *fumS*, *fumT1* and *fumT2* (corresponding to locus tags BKKJ1_2076-2078, including the presumed promoter-containing region located upstream of BKKJ1_2078; this fragment was designated *fumST1T2*) were generated by PCR amplification from *B. kashiwanohense* APCKJ1 using Q5 High-Fidelity Polymerase (New England BioLabs, Herefordshire, UK) and primer pairs 2069pNZ44F and 2069pNZ44R, and 2076-78pCB1.2F and 2076-78pBC1.2R, respectively (primers are listed in Table S4. The resulting *fumA1*-encompassing fragment was digested with KpnI and XbaI, and ligated to the similarly digested pNZ44-*strR*^94^. The ligation mixture was introduced into *E. coli* EC101 by electrotransformation and transformants were selected based on streptomycin resistance. Transformants were checked for plasmid content using colony PCR, restriction analysis of plasmid DNA, and verified by sequencing. Plasmids pNZ44-*fumA1*-*strR* and pNZ44-*strR* were introduced into *E. coli* EC101 harbouring pNZ-M.BbrII-M.BbrIII by electroporation, as previously described^86^, and transformants were selected based on Cm and Strep resistance. Methylation of the plasmid complement of transformants by the M.BbrIII (an isoschizomer of a methylase corresponding to the PstI recognition site) was confirmed by their observed resistance to PstI restriction^86^. Methylated pNZ44-*fumA1*-*strR* was introduced by electrotransformation into *B. breve* UCC2003 and transformants were selected based on streptomycin resistance, generating *B. breve* strain UCC2003-*fumA1*. The *fucST1T2* fragment was digested with XmaI and XbaI, and ligated to similarly digested pBC1.2 to generate pBC1.2-*fucST1T2*. The ligation mixture was introduced into *E. coli* EC101 by electrotransformation and transformants selected based on chloramphenicol resistance. Transformants were checked for plasmid content using colony PCR, restriction analysis of plasmid DNA, and verified by sequencing. Plasmid pBC1.2-*fucST1T2* was introduced into *B. breve* UCC2003-*fumA1* by electrotransformation and transformants were selected based on streptomycin and chloramphenicol resistance, generating strain *B. breve* UCC2003-*fumA1*-*fucST1T2*. In addition, the ‘empty’ cloning vector pBC1.2 was introduced into strain *B. breve* UCC2003-*fumA1* (to generate *B. breve* UCC2003-*fumA1*-pBC1.2), while ‘empty’ cloning vector pNZ44-*strR* was introduced into strain *B. breve* UCC2003-*fucST1T2* (to generate *B. breve* UCC2003-*fumST1T2*-pNZ44-*strR*), in both cases to serve as negative controls. All plasmids and strains are listed in Table S3.

### Sample preparation for NMR analysis

The culture media samples were thawed, vortexed and allowed to stand for 10 min at room temperature. To 540 μl of culture media, 60 μl of a 1.5 M KH_2_PO_4_ buffer (pH 7.4, 100% of deuterium oxide (D_2_O), 2 mM sodium azide and 1% of TSP (3-trimethylsilyl-[2,2,3,3,-^2^H_4_]-propionic acid sodium salt) was added. The mixture was centrifuged at 18,000 × *g* at 4 °C for 5 s. An aliquot of 580 μL of the supernatant was transferred into a 5 mm outer diameter NMR tube. The samples analysed were the cell-free supernatants of *B. kashiwanohense* APCKJ1, wild type *B. breve* UCC2003 or *B. breve* UCC2003-*fumA1*-*fucST1T2* following 24 h growth in mMRS with 0.05% cysteine-HCL and 1% lactose, L-fucose, 2′-FL or 3-FL. Samples of uninoculated media, containing the respective sugars, as well as without any added substrate, were also analysed as controls.

### NMR spectroscopy and data pre-processing

NMR analysis was performed at 300 K on a Bruker 800 MHz spectrometer equipped with a 5 mm CPTCI ^1^H-^13^C/^15^N/D Z-gradient cryoprobe, and automated tuning and matching. ^1^H NMR spectra were acquired using standard one-dimensional pulse sequence, with water presaturation (noesygppr1d sequence) during both the relaxation delay (RD = 4 s) and mixing time (τ_m_ = 10 ms). The receiver gain was set to 16, and acquisition time to 2.73 s for all experiments. Each culture media spectrum was acquired using 4 dummy scans, 32 scans and 64 K data-points with a spectral window set to 20 ppm. Prior to Fourier Transformation, each free induction decay (FID) was multiplied by an exponential function corresponding to a line broadening of 0.3 Hz. ^1^H NMR spectra were manually phased, baseline corrected and digitized over the range δ −0.5 to 11, and imported into MATLAB (2014a, MathWorks, Natick, U.S.). FIDs were referenced to TSP at δ 0.0 ppm. Spectral regions containing residual water (δ 4.69 to 5.18), TSP (δ −0.50 to 0.77), and noise (δ 10.26 to 11.00) were removed prior to probabilistic quotient normalization^95^. Principal component analysis (PCA) was applied to the processed Pareto-scaled NMR data using SIMCA software (v. 14.1; Umetrics, Sweden). This model was validated by a 7-fold cross-validation.

### Identification of metabolites

Metabolites have signals at specific positions in the 1D ^1^H NMR frequency domain spectrum and can have multiple peaks providing a fingerprint for each molecule. The multiplicities, chemical shifts and peak area integration of resonance signals were used for structural identification of metabolites. 2D Homonuclear ^1^H–^1^H Total Correlation SpectroscopY (TOCSY) and ^1^H J-resolved (JRES) experiments were acquired for a representative sample, to confirm the presence of metabolites. TOCSY spectra of selected samples were acquired using the MLEV17 spin-lock scheme and a spectral width of 12 KHz in both dimensions and a mixing time of 60 ms. 32 transients per increment (16 dummy scans) were collected into 452 increments with a relaxation delay of 2 s and acquisition time of 0.34 s. The spectrum was processed into a resolution of 16k (F2) and 1k (F1) using a QSINE function. A JRES spectrum was acquired for the same samples using 8 scans (16 dummy scans) per increment and 80 increments with an acquisition time of 0.41 s, and a relaxation delay of 2 s. JRES spectra were processed using a SINE window function in both dimensions with SSB = 0 and a resolution of 16k (F2) and 256 (F1). The peaks were subsequently tilted by 45**°** and symmetrised. Databases such as the Human Metabolome Data Base (HMDB; http://hmdb.ca/) or the Biological Magnetic Resonance Data Bank (BMRB; http://www.bmrb.wisc.edu) were used for confirmation of assignments. Metabolite concentrations were calculated relative to the CH_3_ signal of alanine which was consistent for all samples and remained unmodified by any of the bacterial strains.

### Bioinformatic analysis

Sequences of proteins identified as upregulated when *B. kashiwanohense* APCKJ1 was grown on 2′-FL as the sole carbon source were used to search for homologs across type strains of all available *Bifidobacterium* species, using BLASTP^71^ at default settings. On-line available genomic data sets of bifidobacteria were first retrieved from the NCBI website (http://www.ncbi.nlm.nih.gov) and aligned using an all-vs-all BLASTP approach^96^, using cut-off values of 50% of similarity across 50% of protein length and a 0.0001 e-value as a significance for the identification of homologous proteins across all available *Bifidobacterium* species. The resulting alignment was subsequently clustered in Markov Cluster Algorithm (MCL) families of orthologous genes using the mclblastline algorithm^96^. The resulting output was used to first build a presence/absence binary matrix, and then the genes of interest were selected and represented in a heatmap employing a code colour grading that represents the degree of sequence similarity, with species ordered by origin of isolation. The products of the genes with locus tags BKKJ1_2072 (designated here as *fumF* and encoding a putative L2-keto-deoxy-fuconate aldolase), BKKJ1_2073 (*fumD* encoding a predicted L-fuconolactone hydrolase), BKKJ1_2074 (*fumC* encoding a putative L-fucose dehydrogenase) and BKKJ1_2075 (*fumE* predicted to specify a L-fuconate dehydratase) were selected as key enzymes of the fucose utilisation pathway in *B. kashiwanohense* APCKJ1. A fucose utilisation cluster was established as present across bifidobacterial genomes when all the four genes *fumF*, *fumD*, *fumC* and *fumE* were found located within the same genomic region with an average similarity of 70% of the four genes at protein level. Automatic protein annotations of the identified clusters were manually checked using the information retrieved from alternative databases such as Pfam (http://pfam.xfam.org) and KEGG (http://www.genome.jp/kegg/). The sequence similarity of these genes and their homologs in bifidobacterial genomes were represented in a heatmap. The identified fucose utilisation clusters identified in *B. longum* subsp. *infantis* ATCC 15697, *B. pseudocatenulatum* DSM 20438 and *B. breve* UCC2003 were directly compared and represented in a locus homology map.

Protein sequences of eighty three GH29 and GH95 α-fucosidases were retrieved from the Cazy (http://www.cazy.org/Glycoside-Hydrolases.html) and GenBank (https://www.ncbi.nlm.nih.gov/genbank) databases. Phylogenetic inference was performed on the eighty three α-fucosidases, with the *E. coli* K-12 DnaA protein (b3702) as an outgroup, using the MEGA7 suite (http://www.megasoftware.net/). Protein sequence alignments were performed using the Muscle module available within MEGA7, and the resulting phylogenetic tree was built using the neighbour joining method based on a statistical assessment of 1000 bootstrap replicates. Finally, the iTOL viewer (https://itol.embl.de) was employed in order to visualize and produce the final tree.

### Ethics approval and consent to participate

The infant faeces used in this study were collected as part of the INFANTMET study cohort. Mothers were approached for consent between February 2012 and May 2014 in Cork University Maternity Hospital, with ethical approval provided by the Cork University Hospital Research Ethics Committee. Ethical approval reference: ECM (w) 07/02/2012.

## Supplementary information

Supplementary Information

## Data Availability

The genome sequence of *B. kashiwanohense* APCKJ1 was deposited at the GenBank under Accession No. CP026729. The microarray data obtained in this study have been deposited in NCBI’s Gene Expression Omnibus database and are accessible through GEO Series Accession Number GSE107439.
