# Author Correction: Metabolism of the predominant human milk oligosaccharide fucosyllactose by an infant gut commensal

**DOI:** 10.1038/s41598-020-73762-1

**Published:** 2020-10-09

**Authors:** Kieran James, Francesca Bottacini, Jose Ivan Serrano Contreras, Mariane Vigoureux, Muireann Egan, Mary O’connell Motherway, Elaine Holmes, Douwe van Sinderen

**Affiliations:** 1 APC Microbiome Ireland, University College Cork, Western Road, Cork, Ireland; 2 School of Microbiology, University College Cork, Western Road, Cork, Ireland; 3 Department of Surgery and Cancer, Imperial College London, South Kensington, London, SW7 2AZ, United Kingdom; 4 The Centre for Computational and Systems Medicine, Health Futures Institute, Murdoch University, Harry Perkins Institute of Medical Research, 5 Robin Warren Drive, Perth, WA 6150 Australia

Correction to: *Scientific Reports* 10.1038/s41598-019-51901-7, published online 28 October 2019

The original version of this Article contained an error in Affiliation 4, which was incorrectly given as ‘Institute of Health Futures, Murdosh University, South Street, Perth, WA, 6150, Australia.’ The correct affiliation is listed below:

The Centre for Computational and Systems Medicine, Health Futures Institute, Murdoch University, Harry Perkins Institute of Medical Research, 5 Robin Warren Drive, Perth, WA 6150, Australia

This error has now been corrected in the HTML and PDF versions of the Article and in the Supplementary Information file that accompanies the Article.

