# Supplementary material for: Metabolism of the predominant human milk oligosaccharide fucosyllactose by an infant gut commensal
Source: Sci Rep. 2019 Oct 28;9:15427. doi: 10.1038/s41598-019-51901-7 (PMC6817895; doi:10.1038/s41598-019-51901-7)
Supplement: Supplementary file 1 — Supplementary Information [file 41598_2019_51901_MOESM1_ESM.pdf]

Metabolism of the predominant human milk oligosaccharide fucosyllactose by an infant gut commensal

Kieran James[a,b,+], Francesca Bottacini[a,+], Jose Ivan Serrano Contreras[c], Mariane Vigoureux[a], Muireann Egan[a,b], Mary O'Connell Motherway[a], Elaine Holmes[c,d] and Douwe van Sinderen[a,b,#]

[a] APC Microbiome Institute and [b] School of Microbiology, University College Cork, Western Road, Cork, Ireland

[c] Department of Surgery and Cancer, Imperial College London, South Kensington, London, SW7 2AZ

[d] The Centre for Computational and Systems Medicine, Health Futures Institute, Murdoch University, Harry Perkins Institute of Medical Research, 5 Robin Warren Drive, Perth, WA 6150, Australia.

[+] These authors contributed equally to this study

[#] Corresponding author

Corresponding author email address: d.vansinderen@ucc.ie

Supplemental Table S1 B. kashiwanohense APCKJ1 genome sequencing reads and quality.

Sequencing Project	Number of Reads	Coverage	Mapped N50 (Long Reads)	Mapped N50 (Short Reads)	Polymerase Read Quality
B. kashiwanohense APCKJ1 Genome	61974	245.02x	17573bp	7971bp	0.853

Supplementary Table S2. *B. longum* subsp. *infantis* ATCC15697 genes involved in the catabolism of fucosyllactose, and their homologs in *B. kashiwanohense* APCKJ1 and *B. breve* UCC2003, based on a blastP search of the APCKJ1 and UCC2003 genomes.

Gene	Predicted Function	Gene	BLASTP Result	Gene	BLASTP Result
Blon_2335	GH95 α-fucosidase	BKKJ1_2069 ***fumA1**$_{kw}$*	77%, 1285, 0.0	Bbr_1288 ***fumA1**$_{br}$*	77%, 1285, 0.0
Blon_2336	GH29 α-fucosidase	BKKJ1_2070 ***fumA2**$_{kw}$*	86%, 879, 0.0	-	-
Blon_2337	L-fucose mutarotase	BKKJ1_2071 ***fumB**$_{kw}$*	87%, 262, 2e-62	-	-
Blon_2306	L-fuconolactone hydrolase	BKKJ1_2073 ***fumD**$_{kw}$*	96%, 513, 0.0	Bbr_1741 ***fumD**$_{br}$*	50%, 248, 7e-87
Blon_2340	L-fuconate dehydratase	BKKJ1_2075 ***fumE**$_{kw}$*	98%, 867, 0.0	Bbr_1744 ***fumE**$_{br}$*	78%, 703, 0.0
Blon_2339	L-2-keto-3-deoxy-fuconate-4-dehydrogenase	BKKJ1_2074 ***fumC**$_{kw}$*	94%, 487, 0.0	Bbr_1743 ***fcuC**$_{br}$*	76%, 390, 6e-143
Blon_2338	L-2-keto-3-deoxy-fuconate aldolase	BKKJ1_2072 ***fumF**$_{kw}$*	89%, 556, 0.0	Bbr_1740 ***fumF**$_{br}$*	70%, 429, 3e-157
Blon_0540	L-1,2-propanediol oxidoreductase	BKKJ1_0429 ***fumG**$_{kw}$*	95%, 750, 0.0	Bbr_1505 ***fumG**$_{br}$*	98%, 765, 0.0

Values in the BLASTP column represent match identity, Bit Score and e-value.
Cut-off values of a minimum Bit Score of 200 bits, a minimum identity of 50% coverage and minimum e-value of 0.0001 were employed.
*Denotes genes not upregulated in transcription during growth on 2-FL or 3-FL.

Supplementary Table S3: Bacterial plasmids and strains used in this work.

Cm^r, Km^r and Strep^r, resistance to chloramphenicol, kanamycin and streptomycin, respectively.
UCC, University College Cork Culture Collection.

**Strain or plasmid**	**Relevant Features (antibiotic resistances are given in brackets)**	**Reference or Source**
Strains		
Escherichia coli strains		
E. coli EC101	Cloning host, repA$^+$ (Km^r)	[1]
E. coli EC101-pNZ-M.BbrII + M.BbrIII	EC101 harbouring pNZ8048 derivative containing *bbrIIM* and *bbrIIIM* (Cm^r)	[2]
E. coli EC101-pBC1.2-fumST1T2	XL1-blue containing pBC1.2-*fumST1T2* (Cm^r)	This study
E. coli EC101-NZ44-fumA1-strR	EC101 harbouring pNZ8048 pNZ44-f*umA1*-(Strep^r)	This study
E. coli EC101-pNZ-M. BbrII + M.BbrIII +pNZ44-*fumA1*-strR	EC101 harbouring pNZ8048 derivative containing *bbrIIM* ,*bbrIIIM* and pNZ44-*fumA1*-(Strep^r)	This study
Lactococcus lactis strains		
L. lactis NZ9000	MG1363, pepN::nisRK, nisin-inducible overexpression host	[3]
L. lactis NZ9700	Nisin-producing strain (Cm^r)	[3]
L. lactis NZ9000-pNZ-*fumA1*	NZ9000 containing pNZ-*fumA1*(Cm^r)	This study
L. lactis NZ9000-pNZ-*fumA2*	NZ9000 containing pNZ-*fumA2* (Cm^r)	This study
L. lactis NZ9000-pNZ44-*fumA1*	NZ9000 containing pNZ44-*fumA1* (Cm^r)	This study
Bifidobacterium sp. Strains		
B. kashiwanohense APCKJ1	Isolate from nursling stool	This study
B. breve UCC2003	Isolate from nursling stool	[4]
B. breve UCC2003-*fumA1*-*fumST1T2*	UCC2003 harbouring pNZ44-*fumA1*-Strep^R and pBC1.2-*fumST1T2* (Cm^r) (Strep^r)	This study
B. breve UCC2003-*fumA1*-pBC1.2	UCC2003 harbouring pNZ44-*fumA1*-Strep^R and pBC1.2 (Cm^r) (Strep^r)	This study
B. breve UCC2003-*fumST1T2*-pNZ44-strR	UCC2003 harbouring pNZ44 -Strep^R and pBC1.2-*fumSP1P2* (Cm^r) (Strep^r)	This study
Plasmids		
pBC1.2	pBC1-pSC101-(Cm^r)	[5]
pBC1.2-*fumST1T2*	(Cm^r), pBC1-pSC101-Cmr harbouring *fumST1T2* and its indigenous promoter	This study
pNZ8150	(Cm^r), nisin inducible translational fusion vector	[6]
pNZ-*fumA1*	(Cm^r), pNZ8150 derivative containing translational fusion of BKKJ_2069 encoding DNA fragment to nisin inducible promoter	This study
pNZ-*fumA2*	(Cm^r), pNZ8150 derivative containing translational fusion of BKKJ_2070 encoding DNA fragment to nisin inducible promoter	This study
pNZ44-strR	(**S**trep^r) pNZ8048 derviative containing constitutive p44 promoter from Lactococcal chromosome, pNZ44, harbouring CNCMI4321_0987	[7]
pNZ44-*fumA1*-strR	(**S**trep^r), pNZ44 harbouring BKKJ_2069 downstream of p44 promoter, and CNCMI4321_0987	This study

Supplementary Table S4: Oligonucleotide primers used in this work.

Purpose	Primer	Sequence (5'-3')
Amplification of the ITS region for *Bifidobacterium* isolate species identification	Bifspp	ggtgtgaaagtccatcgct
	23Sbif	gtctgccaaggcatccacca
Cloning of BKKJ1_2069 in pNZ8150	2069F	tgcatc**cccggg**atgcatcaccatcaccatcaccatcaccatcacaaactcacattcgatggaatc
	2069R	tgcgca**tctaga**cgtaacggatataacgcaatacg
Cloning of BKKJ1_2070 in pNZ8150	2070F	tgcatc**cccggg**atgcatcaccatcaccatcaccatcaccatcacagcaatccaacaaatgatggt
	2070R	tgcgca**tctaga**agtttcatggtgacgtatcgcc
Cloning of 2379bp fragment containing BKKJ1_2069 into pNZ44-strR	2069pNZ44F	ctggtc**ggtacc**ggcgatacgtcaccatgaaact
	2069pNZ44R	tgcgca**tctaga**ataacgcaatacgttaacgccg
Cloning of BKKJ1_2076-2078 in pBC1.2	2076-78pBC1.2F	ctggtc**cccggg**gcccgctgttctctggatg
	2076-78pBC1.2R	tgcgca**tctaga**cgatgcgcttccttctttg

Restriction sites incorporated into oligonucleotide primer sequences are indicated in bold, and His-tag sequences incorporated into nucleotide primer sequences are indicated in italics.

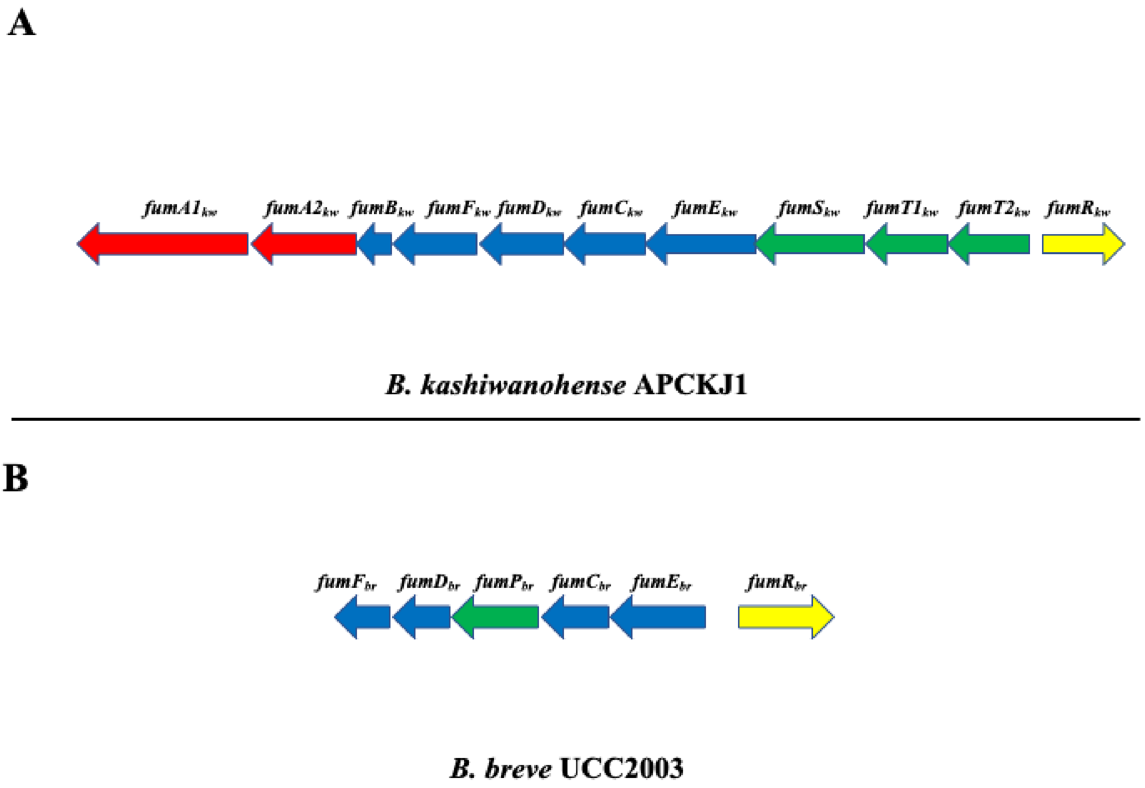

Supplementary Figure S1. Schematic representation of the gene loci involved in the utilisation of: **(A)** 2-FL or 3-FL in *B. kashiwanohense* APCKJ1, and **(B)** L-fucose in *B. breve* UCC2003; as based on transcriptome analysis. The length of the arrows is proportional to the size of the open reading frame and the gene locus name, which is indicative of its putative function, is given inside the arrows. Genes shown in red are predicted to encode proteins with a hydrolytic function, genes shown in yellow are predicted to encode proteins with a regulatory function, genes shown in green are predicted to encode proteins with a transport function and genes shown in blue are predicted to encode proteins with another metabolic function. Figure adapted from thesis Figure 5.3; James, 2018 [8].

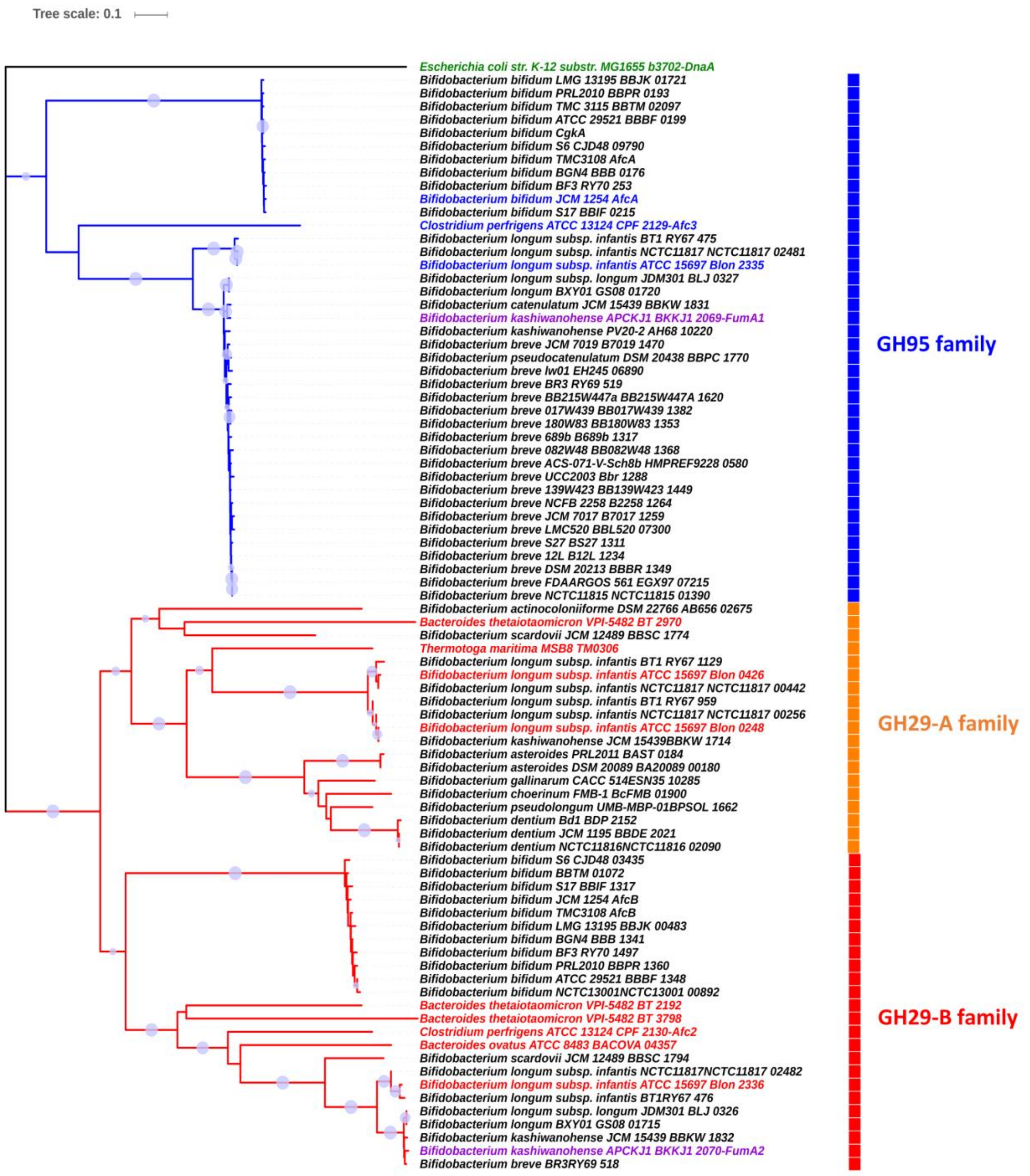

Supplementary Figure S2. Phylogenetic analysis of GH29 anf GH95 α-fucosidases. Neighbour-joining tree based on the alignment of eighty three α-fucosidases retrieved from the Cazy database (http://www.cazy.org/Glycoside-Hydrolases.html). Previously characterized α-fucosidases are highlighted in blue (GH95) and red (GH29), while the FumA1 and FumA2 proteins from the current study are highlighted in purple. Light blue circles indicate bootstrap values higher than 70 %, while the outgroup sequence is highlighted in green**.**

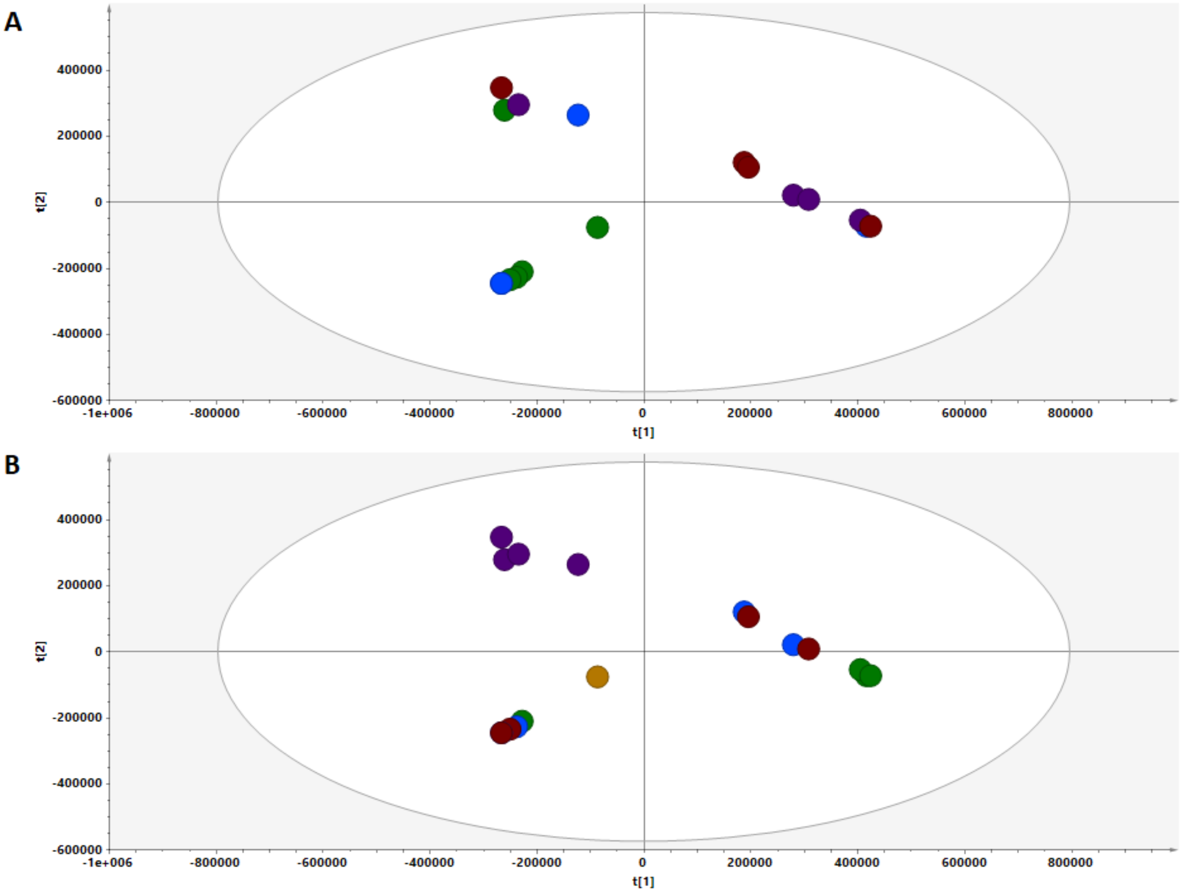

Supplementary Figure S3: PCA plot depicting the relative similarity of the metabolite contents of the samples analysed by NMR; coloured by **(A)** bacterial species, and **(B)** sugar added to culture media.

R2X = 59.5% Q2 = 45.8%.

(A) Uninoculated media (green), *B. breve* UCC2003 WT (blue), *B. breve* UCC2003 *fumA1-fumST1T2* (red), *B. kashiwanohense* APCKJ1 WT (purple).

(B) No sugar added (orange), 1% lactose (green), 1% L-fucose (purple), 1% 2'-FL (blue), 1% 3-FL (red).

References

1. Law J, Buist G, Haandrikman A, Kok J, Venema G, Leenhouts K: **A system to generate chromosomal mutations in Lactococcus lactis which allows fast analysis of targeted genes.** *J Bacteriol* 1995, **177:**7011-7018.
2. O'Connell Motherway M, O'Driscoll J, Fitzgerald GF, Van Sinderen D: **Overcoming the restriction barrier to plasmid transformation and targeted mutagenesis in Bifidobacterium breve UCC2003.** *Microb Biotechnol* 2009, **2:**321-332.
3. de Ruyter PG, Kuipers OP, de Vos WM: **Controlled gene expression systems for Lactococcus lactis with the food-grade inducer nisin.** *Applied and Environmental Microbiology* 1996, **62:**3662-3667.
4. Maze A, O'Connell-Motherway M, Fitzgerald GF, Deutscher J, van Sinderen D: **Identification and characterization of a fructose phosphotransferase system in Bifidobacterium breve UCC2003.** *Appl Environ Microbiol* 2007, **73:**545-553.
5. Alvarez-Martin P, O'Connell-Motherway M, van Sinderen D, Mayo B: **Functional analysis of the pBC1 replicon from Bifidobacterium catenulatum L48.** *Appl Microbiol Biotechnol* 2007, **76:**1395-1402.
6. Mierau I, Kleerebezem M: **10 years of the nisin-controlled gene expression system (NICE) in Lactococcus lactis.** *Appl Microbiol Biotechnol* 2005, **68:**705-717.
7. Bottacini F, Morrissey R, Esteban-Torres M, James K, van Breen J, Dikareva E, Egan M, Lambert J, van Limpt K, Knol J, et al: **Comparative genomics and genotype-phenotype associations in Bifidobacterium breve.** *Sci Rep* 2018, **8:**10633.
8. James K: **Metabolism of human milk oligosaccharides by infant-associated bifidobacteria**
   Doctoral. University College Cork, Microbiology; 2018.
